# A recombinant humanized type III collagen coating with anti-inflammatory and endothelialization-promoting effects for left atrial appendage closure devices

**DOI:** 10.1093/rb/rbag114

**Published:** 2026-06-05

**Authors:** Yihang Zhou, Jiaqi Wang, Longjian Zhang, Li Yang, Yunbing Wang

**Affiliations:** National Engineering Research Center for Biomaterials, College of Biomedical Engineering, Sichuan University, Chengdu 610065, China; National Engineering Research Center for Biomaterials, College of Biomedical Engineering, Sichuan University, Chengdu 610065, China; National Engineering Research Center for Biomaterials, College of Biomedical Engineering, Sichuan University, Chengdu 610065, China; National Engineering Research Center for Biomaterials, College of Biomedical Engineering, Sichuan University, Chengdu 610065, China; National Engineering Research Center for Biomaterials, College of Biomedical Engineering, Sichuan University, Chengdu 610065, China; Research Unit of Minimally Invasive Treatment of Structural Heart Disease, Chinese Academy of Medical Sciences, Beijing 100730, China; Chengdu Minshan Institute of Biomaterials, Chengdu 610200, China

**Keywords:** left atrial appendage occluder, recombinant humanized collagen type III, reendothelialization, anti-inflammatory

## Abstract

Transcatheter cardiac occluders represent a mainstay of minimally invasive therapy for congenital heart disease and for the prevention of left atrial appendage-related thrombosis. However, their long-term clinical efficacy is constrained by frequent post-implant complications, including device-related thrombosis, persistent inflammation, incomplete reendothelialization, and delayed tissue healing. Herein, we developed a hierarchical functional modification strategy for biodegradable polymers via stable covalent grafting of recombinant humanized type III collagen (rhCol III)—a biomaterial with robust cell adhesiveness and low immunogenicity—onto the polymer surface. Systematic *in vitro* assays demonstrated that the rhCol III coating effectively inhibited coagulation, enhanced endothelial and cardiomyocyte function, and modulated inflammation through promoting the polarization of macrophages to the anti-inflammatory M2 phenotype. *In vivo*, this innovative coating achieved 71.68% endothelialization at 40 days post-implantation, nearly double that of unmodified controls, and transcriptomic analysis further validated its acceleration of reendothelialization and tissue healing. Collectively, this multifunctional rhCol III coating integrates anticoagulant, anti-inflammatory and pro-endothelialization properties, providing a promising translational strategy to advance left atrial appendage occluders from passive mechanical occlusion to active biofunctional regeneration.

## Graphical Abstract

**Graphical Abstract:**
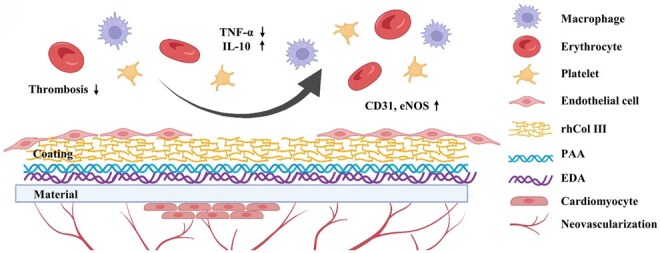

## Introduction

Cardiovascular diseases (CVDs) still rank as the top contributor to worldwide disease burden. World Health Organization (WHO) data show that as early as 2019, CVD-related deaths accounted for one-third of all deaths globally, and this upward trend has persisted [1–3]. As the most common sustained arrhythmia, atrial fibrillation (AF) is a core driver of the high morbidity and mortality linked to CVDs [4, 5]. Owing to the unique anatomical features of the left atrial appendage (LAA), this structure is particularly prone to thrombus formation during AF episodes [6], rendering it the primary source of thrombosis in patients with AF.

Clinical evidence demonstrates that over 90% of thrombi causing ischemic stroke in AF patients originate from the LAA [7, 8]. The risk of ischemic stroke is five times higher among AF patients than healthy civilians [9], with a post-stroke disability rate of up to 70% [10]. More critically, the overall prevalence of AF in China has reached 1.6%, and the total number of patients is projected to exceed 20 million by 2050 due to population aging [11]. Consequently, preventing and controlling LAA thrombosis-related stroke has become an urgent public health priority in the cardiovascular field in China [12].

Against this clinical backdrop, transcatheter left atrial appendage closure (LAAC) has emerged as a targeted therapy [13–15]. LAAC involves implanting an occluder to eliminate the thrombotic source of the LAA. Superior to traditional open surgery, this minimally invasive method causes slighter tissue trauma and enables faster postoperative recovery. It has thus become a critical therapeutic option for AF patients at high bleeding risk or those ineligible for long-term anticoagulation, significantly improving clinical outcomes in this population [16]. The core goal of LAAC is to reduce stroke risk. However, as a foreign body, the implanted occluder carries early complication risks, and poor biocompatibility at the tissue-material interface remains a major constraint on long-term clinical efficacy. Delayed endothelialization, device-related thrombosis (DRT), and chronic inflammation can trigger severe complications such as arrhythmia and cerebral infarction [15, 17]. Therefore, tuning the surface biointerface properties of left atrial appendage occluders (LAAOs) to accelerate endothelialization, inhibit coagulation, and modulate inflammatory responses constitutes the core challenge for enhancing the clinical safety and efficacy of LAAC [16, 18, 19].

Accordingly, the development of functional surface coatings for LAAOs is a key strategy to optimize post-implant performance [20]. The core goal is to achieve synergistic effects of rapid endothelialization, potent anticoagulation, and mild inflammatory response by modulating the device’s surface biointerface properties. Previous studies have shown that incorporating diverse functional components onto device surfaces via coating technology—including anticoagulant coatings [21], apixaban drug-loaded coating [22] and bioactive motifs such as arginine–glycine–aspartic acid peptide motifs [23] and nitric oxide (NO) donors [24]—can improve therapeutic outcomes [25, 26]. Despite these advancements, existing coatings still have inherent limitations, including monofunctionality, poor *in vivo* stability, and insufficient biosafety, which hinder their ability to meet the complex clinical requirements of LAA intervention.

Biocompatibility, low immunogenicity and bioactivity are core prerequisites for cardiovascular implant coating materials [27]. Currently, widely used plasma protein coatings (e.g. albumin, fibrin) only act as passive barriers to reduce acute thrombosis, lacking active bioactivity to resolve delayed endothelialization and long-term restenosis [28–32]. The mainstream animal-derived type I collagen (Col I), despite good film-forming property, is limited by excessive intimal hyperplasia risk and inherent safety defects of animal-derived materials [33, 34].

In contrast, recombinant human type III collagen (rhCol III) precisely replicates the structure and biological function of native human Col III, a core component of the vascular endothelial basement membrane [35–38]. It fundamentally eliminates the safety flaws of animal-derived collagen and possesses a unique active capacity to promote endothelialization, regulate vascular regeneration and tissue homeostasis—bioactivity completely absent in albumin coatings and Col I [39, 40]. These merits make rhCol III the optimal biomaterial for the functional coating construction in this study [36–38, 41].

Most current cardiovascular implant coatings are fabricated via non-covalent methods, such as physical adsorption [42]. Such coatings exhibit weak interfacial adhesion to device substrates and are prone to delamination under *in vivo* blood shear stress and dynamic tissue motion, leading to rapid loss of biofunction [43]. To address this critical limitation, this study employed covalent crosslinking to fabricate a robust, customized rhCol III coating, where strong covalent bonding ensures stable coating-substrate adhesion and long-term structural integrity *in vivo* [44].

Notably, the anticoagulant performance of the coating is critical, and relying solely on collagen to achieve anticoagulation has limited efficacy [45]. Furthermore, the biodegradable polymer substrate is inherently inert [36]. Therefore, an intermediate interfacial layer is required to provide sufficient reactive sites for collagen grafting, as well as to impart anticoagulant properties, thereby improving the hemocompatibility of the coating. Poly(acrylic acid) (PAA) with well-validated intrinsic anticoagulant activity has been widely used in cardiovascular functional coatings, as its abundant carboxyl anions repel pro-coagulant components via electrostatic interaction to enhance surface hemocompatibility [46–48].

On this basis, ethylenediamine (EDA) coupled with PAA was selected as the intermediate layer. EDA modification introduces abundant primary amine groups onto the surface of the biodegradable material [46], and the subsequent covalent reaction between the amine-functionalized surface and PAA introduces numerous carboxyl groups, which afford sufficient active sites for further collagen grafting [38, 46]. Accordingly, we fabricated an EDA–PAA/rhCol III coating on biodegradable cardiac occluder surfaces via stepwise covalent grafting. With EDA–PAA as the anticoagulant bridging interlayer and rhCol III as the core bioactive molecule, this coating achieves synergistic anticoagulant, anti-inflammatory and pro-endothelialization effects, providing a novel modification strategy to improve the *in vivo* safety and long-term performance of biodegradable cardiac occluders.

The study systematically evaluated the physicochemical stability, hemocompatibility, and cell-regulating capacity of the EDA–PAA/rhCol III coating through *in vivo* and *in vitro* experiments. The coating shows favorable effects on improving cardiac occluder function, as supported by the results. To address core clinical issues such as DRT and delayed endothelialization following LAAC procedures [49], this work aimed to provide a safe, efficient, and universal technical solution for LAAO surface modification, thereby promoting the clinical translation of LAAC therapy from physical occlusion to biological functional regeneration.

## Materials and methods

### Materials and reagents

Poly(p-dioxanone) (PDO) sheets and monofilaments were provided by Shanghai Shape Memory Alloy Co., Ltd. (Shanghai, China). RhCol III was obtained from Shanxi Jinbo Bio-Pharmaceutical Co., Ltd. (Shanxi, China). Fluorescein isothiocyanate (FITC), EDA, PAA, 1-(3-dimethylaminopropyl)-3-ethylcarbodiimide hydrochloride (EDC), N-hydroxysuccinimide (NHS), Cell Counting Kit-8 (CCK-8), phosphate-buffered saline (PBS), propidium iodide (PI) and fluorescein diacetate (FDA) were all purchased from Sigma-Aldrich (Shanghai, China). All experiments were carried out with ultrapure water purified by a Millipore purification system.

### Preparation of coatings

To ensure the reliability of data collection, the same coating was applied to multiple substrates, namely, polylactic acid (PLA) and PDO, for subsequent material and biological characterization. Specifically, the substrates were rapidly immersed in a freshly prepared EDA solution (40% v/v, dissolved in ultrapure water, UPW) for 12 h and then rinsed with UPW. The cleaned sheets were then soaked in a 1:1 mixed solution of PAA (4 mg/mL, dissolved in UPW) and EDC/NHS (4 mg/mL, dissolved in PBS) at 25°C for 8 h. Afterward, the sheets were rinsed with UPW to remove poorly adsorbed components. The sheets were subsequently placed in an EDC/NHS solution (10 mg/mL, dissolved in PBS) for 4 h. After removal, the substrates were immersed in recombinant human collagen type III (rhCol III) solution (1 mg/mL, dissolved in PBS) for 8 h and thoroughly rinsed with UPW. The final product was designated EDA–PAA/rhCol III. Uncoated substrates served as the control group.

Given the advantages of PLA, such as high purity of commercial preparations, homogeneous surface properties, and easy availability, it was mainly used for physicochemical characterization of the coating to accurately verify the preparation efficiency and physicochemical stability of the EDA–PAA/rhCol III coating. In contrast, PDO, a commonly used material for the skeleton of clinical cardiac occluders, was primarily employed for biocompatibility and *in vitro*/*in vivo* functional verification experiments, including cytotoxicity, hemocompatibility, and intravascular implantation tests. These experiments aimed to evaluate the biological function and safety of the coating using a model closer to clinical needs. Preliminary experiments confirmed that there were no significant differences in the grafting efficiency or surface chemical composition of the same coating on the PLA and PDO surfaces and that the consistency of the coating itself was not affected by the substrate.

### Coating characterization

Surface morphology was measured via field-emission scanning electron microscopy (FE-SEM; Apreo S HiVac, Thermo Fisher Scientific, USA). Surface roughness was determined using atomic force microscopy (AFM; MFP-3D-BIO, Asylum Research, USA). The chemical structure and elemental composition of the coating were determined by attenuated total reflection Fourier transform infrared (ATR-FTIR) spectroscopy (Spectrum TWO, PerkinElmer, USA) and X-ray photoelectron spectroscopy (XPS, AXIS Ultra DLD, Kratos, UK). Besides, surface hydrophilic performance and zeta potential were measured separately. Water contact angle (WCA) was characterized with Attension Theta (Biolin Scientific, Sweden), and the SurPASS 3 analyzer (Anton Paar, Austria) served for solid-surface zeta potential analysis.

### Coating stability test

In order to examine the stability of the coating material, we prepared rhCol III conjugated with FITC (Supplementary material S1), and the FITC-labeled EDA–PAA/rhCol III coating was constructed following the aforementioned method [32]. The substrates with FITC-labeled EDA–PAA/rhCol III coating were immersed in PBS, with the PBS replaced every 2 days, and incubated on a shaker at 75 rpm and 25°C in the dark. Fluorescence intensity changes of coatings submerged in PBS were detected at three time points (0, 7, 14 days) by the ZEISS LSM880 confocal laser scanning microscope. ImageJ was applied to perform quantitative statistics, and the baseline fluorescence intensity measured on day 0 was standardized to 100%.

### Static platelet and whole-blood adhesion

Fresh whole rabbit blood was mixed with sodium citrate at a volume ratio of 9:1, and the supernatant was collected by centrifugation at 1500 rpm for 15 min to obtain platelet-rich plasma (PRP). Afterward, bare, EDA, EDA–PAA, and EDA–PAA/rhCol III-coated PDO sheets (thickness: 500 μm) were incubated with PRP and whole blood at 37°C for 1 h. After the samples were rinsed with PBS and fixed in 2.5% glutaraldehyde for 12 h, the platelets and RBCs attached to the surface were observed using SEM. ImageJ software combined with lactate dehydrogenase (LDH) assay kits was used for platelet counting and quantitative evaluation (Supplementary material S2) [50].

### *In vitro* blood circulation test

Ethical authorization for all experiments was obtained from the Medical Ethics Committee of Sichuan Provincial Administration of Laboratory Animals. The whole study was implemented following relevant guidelines on laboratory animal care and utilization formulated by Sichuan University (KS2020394). Male New Zealand white rabbits (weight: 2.8–3.0 kg; provided by Chengdu Duosi Co., Ltd.) were used to develop an arteriovenous shunt model to perform *in vitro* blood circulation tests [36, 51]. Under anesthetic conditions, the cervical arteries and veins of rabbits were dissected free before linking these blood vessels with standard PVC catheters. A circulation loop was formed by closely attaching sterile PDO sheets to the inner wall, and the circulation was maintained for 2 h. All specimens were harvested and washed with PBS. The occlusion degree of catheters was analyzed using ImageJ, and surface microscopic features were characterized by SEM after specimen fixation and dehydration.

### Hemolysis test

Rabbit whole blood samples underwent 15-min centrifugation at 1500 rpm. The precipitated red blood cells (RBC) were gathered and diluted 50-fold with PBS to obtain a 2% RBC working solution. Four types of PDO specimens including bare substrates, EDA, EDA–PAA and EDA–PAA/rhCol III-coated materials were co-cultured with the prepared RBC solution. Ultrapure water-diluted RBCs were defined as positive control group (+), and PBS-diluted RBCs were taken as negative control group (−). All mixtures were subjected to centrifugation at 3000 rpm for 5 min after incubation, and the absorbance of the resulting supernatants was tested at 540 nm by a microplate reader. The hemolysis rate was calculated by the following formula:


Hemolysis rate (%)=(ODsample-ODnegative)/(ODsample-ODnegative)×100%

### Cytotoxicity test

Bare PDO, EDA, EDA–PAA, and EDA–PAA/rhCol III-coated PDO sheets (1 cm × 1 cm) were placed in 24-well cell culture plates. L929 cells and human umbilical vein endothelial cells (HUVECs) (1 mL; Otwo Biotech, Shenzhen, China) were subsequently seeded at a density of 2 × 10^4^ cells/mL. Held at 37°C with 5% carbon dioxide, cells were maintained for 24 h in ScienCell culture medium containing 10% FBS and 1% penicillin-streptomycin (PS), after which they were subjected to live/dead staining to observe cell viability. The details were described in Supplementary material S3.

### Evaluation of human umbilical vein endothelial cell behavior

Endothelial cell medium supplied by ScienCell Research Laboratories (USA) was used to culture HUVECs purchased from Otwo Biotech (Shenzhen, China). The medium contained 10% FBS and 1% PS, with the culture environment set at 37°C with 5% carbon dioxide. We assessed the survival, proliferation, migration and tube formation of HUVECs, with detailed methods provided in Supplementary material S4.

### Evaluation of H9c2 cell behavior

Dulbecco’s modified Eagle’s medium (DMEM; Gibco, Thermo Fisher Scientific, USA) was applied for the cultivation of H9c2 rat embryonic cardiomyoblasts obtained from Procell (Wuhan, China). The culture medium was supplemented with 10% fetal bovine serum (FBS) and 1% PS, and cells were incubated under a 5% carbon dioxide atmosphere. The viability, proliferative capacity, and morphology of H9c2 cells were assessed, with detailed procedures described in Supplementary material S5.

### Evaluation of *in vitro* macrophage behavior

Mouse monocyte-macrophage leukemia cells belonging to the RAW 264.7 line, provided by Xihua Hospital (Chengdu, China), were grown in high-glucose DMEM medium (Gibco, Thermo Fisher Scientific, USA) supplemented with 10% FBS and 1% FBS substitute at 37°C with 5% carbon dioxide. The viability, proliferation ability, morphological characteristics and expressions of tumor necrosis factor-α (TNF-α) and transforming growth factor-β1 (TGF-β1) of the RAW 264.7 cells were investigated (Supplementary material S6).

### *In vivo* subcutaneous implantation test

Subcutaneous implantation assays were conducted on male Sprague–Dawley rats (weight: 200–220 g; provided by Chengdu Dossy Co., Ltd.) to explore inflammatory responses inside living bodies [36, 51]. Under anesthesia, sterile bare, EDA, EDA–PAA and EDA–PAA/rhCol III-coated PDO sheets (1 cm in diameter, circular and 1 mm in thickness) were implanted into the dorsal region of the rats (two sheets on each side). After 15 and 30 days, the proliferative fibrous tissue surrounding the samples was collected and subjected to hematoxylin and eosin (H&E) staining (G1005; Servicebio, Wuhan, China) and immunofluorescence staining with an anti-IL-10 antibody (GB11108; Servicebio, Wuhan, China) and anti-TNF-α antibody (GB11188; Servicebio, Wuhan, China). The thickness of the fibrous capsule and the relative density of IL-10 and TNF-α were calculated using ImageJ software (with the control group set as 100%).

### Intravascular implantation test of PDO monofilaments

PDO monofilament intravascular implantation was performed on male New Zealand white rabbits weighing 2.4–2.6 kg, aiming to replicate the direct contact state of the occluder scaffold with endocardium tissue. Under anesthesia, sterilized bare PDO monofilaments, as well as EDA, EDA–PAA and EDA–PAA/rhCol III-coated PDO monofilaments (300 μm in diameter and 20 mm in length), were implanted into the carotid arteries of the rabbits and sutured to the inner wall of the blood vessels. After 20 and 40 days, blood vessel samples containing PDO monofilaments were collected. The growth of endothelial cells on the monofilament surface was observed via immunofluorescence co-staining using anti-CD31 antibody (Abcam, UK) and anti-endothelial NO synthase (eNOS) antibody (Abcam, UK). To further evaluate tissue biocompatibility and endothelialization progression, section samples were processed with H&E staining and anti-CD68 immunohistochemical staining (Invitrogen, Carlsbad, CA, USA). Anti-CD31 antibody staining was additionally applied to quantify the degree of endothelial coverage. The relevant statistical data were analyzed using ImageJ software, with the carotid arteries of healthy rabbits used as the reference (set to 100%).

### Transcriptome sequencing

Transcriptome sequencing (RNA-seq) was performed to analyze the RNA transcription profiles of HUVECs extracted after 72 h of culture on different material surfaces, aiming to explore the mechanism by which samples affect cell phenotype and function at the genetic level. Specifically, 4 mL of a HUVEC suspension at a density of 4 × 10^4^ cells/mL was seeded onto bare PDO sheets and EDA–PAA/rhCol III-modified PDO sheets (2 cm × 2 cm). When the cell confluence on the sample surface reached approximately 85%, total RNA was extracted from the cells on the sample surface using TRIzol reagent. The integrity of the RNA was evaluated by denaturing gel electrophoresis, and RNA quantification and quality inspection were performed using a Nanodrop 1000.

After passing RNA quality control, RNA was transcribed into complementary DNA (cDNA) using reverse transcriptase, followed by polymerase chain reaction amplification and purification to construct a cDNA library. The library was loaded onto the Illumina HiSeq 4000 sequencing platform, and RNA-seq was initiated using the TruSeq SR Cluster Kit v3-cBot-HS reagents. After sequencing, the fluorescence image data were analyzed using Solexa pipeline version 1.8 software, and the sequencing data quality was evaluated using FastQC software.

The sequencing data that passed quality control were subjected to differentially expressed gene (DEG) analysis using the Ballgown R package, with the screening criteria of fold change >1.2 and *P-*value < 0.05. On this basis, Gene Ontology (GO) analysis and the Kyoto Encyclopedia of Genes and Genomes (KEGG) database were further used for in-depth analysis of the RNA-seq data, and the results were visualized using topGO software.

### Statistical analysis

All experiments were performed in at least three independent replicates. Data analysis was conducted using ImageJ and Origin 2021 software. All experimental data underwent independent blinded analysis by two researchers, with results expressed as mean ± standard deviation (SD). Bartlett’s test was applied to verify the homogeneity of group variances. Inter-group differences for two groups were analyzed by an independent-samples *t*-test; for multi-group comparisons, a one-way ANOVA was conducted, followed by Tukey’s post hoc test for multiple comparisons. Different superscript letters denote significant between-group differences (*P *< 0.05), while the same letter denotes no significant difference (*P *> 0.05). Error bars indicate SD.

## Results and discussion

### Preparation and characterization of the functional recombinant humanized type III collagen coating

**Figure 1:**
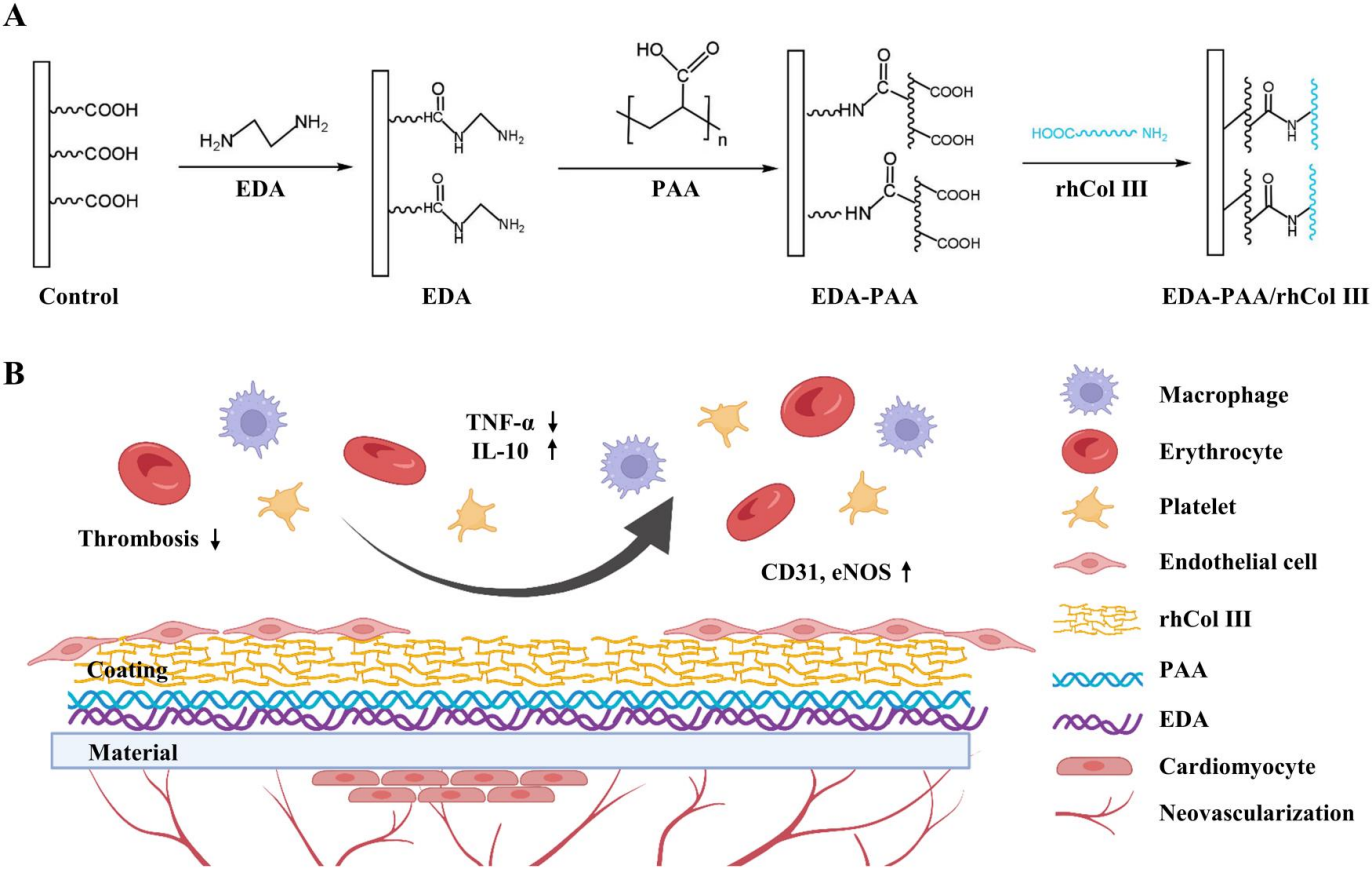
Schematic of preparation and functional model of rhCol III collagen coating. (**A**) The EDA–PAA/rhCol III coating is created by covalently crosslinking EDA with PAA and then grafting rhCol III onto the surface. (**B**) EDA–PAA/rhCol III coating promotes the adhesion and proliferation of endothelial cells (ECs) and inhibits the polarization of macrophages toward the proinflammatory M1 phenotype.

The preparation and physicochemical characterization of the functional recombinant humanized type III collagen coating (EDA–PAA/rhCol III) were carried out using PLA as the substrate (see “Preparation of coatings” section for the selection basis) [52]. The high purity and surface uniformity of PLA minimize substrate-related interference in the characterization results, enabling a more accurate reflection of the structure and performance of the coating [53]. The preparation process involved three-step covalent grafting (Figure 1). First, EDA was used as a crosslinking agent, and it underwent a condensation reaction with carboxyl (–COOH) groups on the surface of the degradable biomaterial under mild conditions [54], resulting in an EDA coating. Subsequently, PAA was introduced via the EDC/NHS activation system, and the density of reactive –COOH groups on the material surface was significantly increased through covalent grafting (designated EDA–PAA) [55]. Finally, the –COOH groups on PAA and the amino groups (–NH_2_) in rhCol III molecules underwent covalent condensation mediated by EDC/NHS activation, forming stable amide bonds (–CO–NH–) [56]; in this way, efficient and stable loading of rhCol III was achieved, and the EDA–PAA/rhCol III coating was ultimately obtained [32, 36].

Notably, compared with direct grafting of rhCol III onto pristine biodegradable polymeric substrates, the three-step covalent grafting strategy proposed in this work featured a rationally designed hierarchical architecture and exhibited significant multifaceted advantages. Specifically, the EDA amination modification in the first step provided abundant reactive amino sites on the surface of inert PLA or PDO substrates, laying a structural foundation for the subsequent stable grafting of the multi-layer coating. The PAA intermediate layer in the second step simultaneously overcame two critical limitations of the pristine substrate and direct grafting strategy. The inherent hydrophobicity of pristine PLA/PDO substrates tends to trigger non-specific protein adsorption, and native rhCol III itself lacks intrinsic anticoagulant activity. The grafted PAA endows the coating with intrinsic anticoagulant properties via chelating blood calcium ions and inhibiting platelet adhesion [57], while providing a large number of carboxyl active sites for the controllable immobilization of the outermost bioactive layer. Furthermore, the covalently grafted rhCol III at the outermost layer of the coating constructs a highly biocompatible and cell-friendly interface, which not only promotes endothelial cell adhesion and proliferation by virtue of its excellent biocompatibility [58] but also elicits anti-inflammatory impacts by mediating macrophage polarization to the anti-inflammatory M2 subtype [36].

Throughout the stepwise grafting process, a three-level amide bond network was formed within the system, which achieved high loading capacity and stable immobilization of rhCol III, and fundamentally resolved the key bottlenecks of easy coating exfoliation and low loading efficiency inherent to direct grafting approaches. Collectively, this hierarchical design enabled the successful construction of a multifunctional coating system integrating anticoagulant, anti-inflammatory, and pro-endothelialization functions, which can fully satisfy the clinical application requirements after LAAO implantation [59].

**Figure 2:**
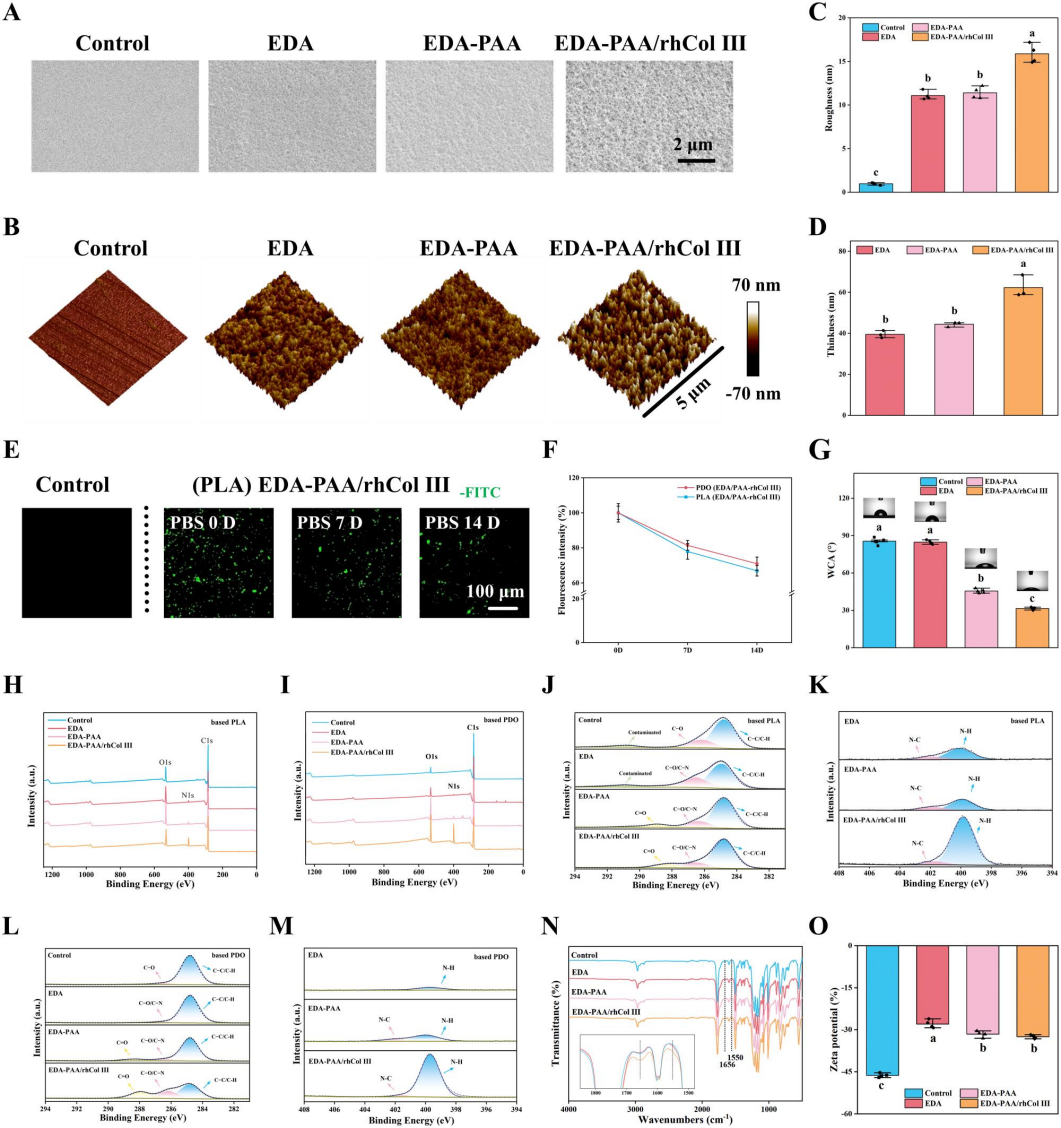
Characterization of collagen coatings on PLA substrates. (**A**) SEM and (**B**) AFM images of a bare substrate and substrates with EDA, EDA–PAA, and EDA–PAA/rhCol III coatings. (**C**) Surface roughness and (**D**) thickness of bare substrates and substrates with EDA, EDA–PAA and EDA–PAA/rhCol III coatings. (**E**) Quantitative analysis of the fluorescence intensity and (**F**) rhCol III density of coatings after they were soaked in PBS for 0, 7 and 14 days. (**G**) Surface hydrophilicity of different samples. (**H** and **I**) XPS survey spectra of PLA and PDO substrates with no coating and with EDA, EDA–PAA and EDA–PAA/rhCol III coatings, respectively. (**J**–**M**) High-resolution XPS C1s spectra, and high-resolution XPS N1s spectra of PLA and PDO substrates with no coating and with EDA, EDA–PAA and EDA–PAA/rhCol III coatings, respectively. (**N**) ATR-FTIR spectra and (**O**) surface zeta potential.

To verify the successful preparation of the EDA–PAA/rhCol III coating and confirm the rationality of the above design, we systematically characterized the surface morphology, chemical composition and physicochemical properties of the coatings at each grafting step. The surface morphologies of the bare substrate, EDA coating, EDA–PAA coating and EDA–PAA/rhCol III coating were observed using FE-SEM, energy dispersive X-ray spectroscopy and AFM. As shown in Figure 2A and B and Supplementary Table S1, the substrate gradually reacted with EDA and PAA to form a surface coating, followed by the grafting of collagen onto the EDA–PAA coating. With the introduction of rhCol III, the surface roughness and thickness of the EDA–PAA/rhCol III coating increased (Figure 2C and D), indicating successful grafting of rhCol III.

FITC was specifically conjugated to rhCol III through a stable thiourea covalent bond with excellent hydrolytic stability in neutral PBS [60]. Post-labeling ultrasonication for 5 min was performed to completely remove unbound and loosely bound free fluorophores, ensuring all detected fluorescence signals originated exclusively from FITC stably conjugated to rhCol III. The acquired FITC-tagged rhCol III was incorporated into coatings to evaluate stability performance. As shown in Figure 2E and F, homogeneous green fluorescence appeared on the surface of the newly fabricated FITC-labeled EDA–PAA/rhCol III coating surface. After 7 days of PBS immersion, the sample retained 76.08 ± 6.49% of its initial fluorescence intensity, and 60.93 ± 5.35% of the signal was still maintained after 14 days. Although collagen peptide cleavage may reduce FITC signal while residual peptides remain bioactive, the gradual time-dependent decline in fluorescence signal showed typical enzymatic degradation kinetics, which can partially reflect the integrity and retention of collagen on the coating surface. These results confirmed that the EDA–PAA/rhCol III coating has sufficient stability to exert its effects within the first two weeks post-implantation. Furthermore, WCA measurements (Figure 2G) indicated that material hydrophilicity was significantly enhanced after PAA and rhCol III modification.

XPS (Figure 2H and I) revealed that C, O and N signals were identified in all three coatings deposited on the PLA and PDO substrates, with the N signal being significantly increased in the EDA–PAA/rhCol III coating. High-resolution C1s and N1s spectra of the EDA–PAA/rhCol III-coated samples on different substrates were subjected to peak-fitting analysis, as illustrated in Figure 2J–M. The incorporation of PAA and recombinant human collagen type III (rhCol III) led to the emergence of a C=O peak at approximately 288.4 eV in the coating, and the C=O peak intensity was markedly increased in the EDA–PAA/rhCol III coating. Moreover, the introduction of EDA resulted in the presence of N on the coating surface, and the incorporation of rhCol III increased the relative proportion of the N–H peak in the EDA–PAA/rhCol III coating. Elemental ratio analysis of the two substrate materials (Supplementary material S1) revealed no significant differences in chemical composition or stable elemental proportions of the surface coatings on both PLA and PDO substrates after modification with the EDA–PAA/rhCol III coating. After immersion in PBS for 7, 14, and 28 days, the results of the analysis on the XPS survey scan spectra and surface elemental composition ratios of the substrates, which were modified with different coatings, showed that the N content on the material surface remained stable (Supplementary material S2). Collectively, these results confirmed that the EDA–PAA/rhCol III coating could be successfully fabricated on both substrates and could exhibit good stability.

ATR-FTIR was adopted to analyze the chemical composition of coating samples (Figure 2N). In the EDA, EDA–PAA and EDA–PAA/rhCol III coatings, at 1656 and 1550 cm^−1^, the characteristic peaks assigned to the amide I (C=O stretching) and amide II (N–H bending) bands of amide moieties appeared, respectively. This phenomenon indicated the presence of amide groups in all these materials. Notably, the C=O stretching vibration peak and N–H bending vibration peak were more pronounced for the EDA–PAA/rhCol III coating, implying that the introduction of rhCol III increased the concentration of amide groups in the coating [61].

The zeta potential results for the solid surfaces (Figure 2O) revealed that the introduction of EDA increased the surface zeta potential and decreased the electronegativity of the material. With the subsequent introduction of PAA and rhCol III, the number of carboxyl groups in the coating increased, leading to increased electronegativity and decreased zeta potential. These results further confirmed the successful preparation of the EDA, EDA–PAA and EDA–PAA/rhCol III coatings.

### Hemocompatibility of the collagen coating

Hemocompatibility is the core evaluation index for cardiovascular implantable devices [62]. The experiments in this section were conducted using PDO as the substrate; PDO is the mainstream scaffold material for clinically degradable occluders, and its surface properties (e.g. degradation rate and mechanical strength) are consistent with the actual application scenarios of occluders. When PDO is used as the control, the *in vivo* anticoagulant effect of the coating can be simulated with maximal accuracy.

The adhesion, aggregation, activation and deformation of blood cells in contact with the material surface are closely related to thrombus formation [62]. The hemocompatibility of the bare, EDA, EDA–PAA and EDA–PAA/rhCol III coatings was evaluated through static platelet/whole blood adhesion, a rabbit *ex vivo* blood circulation model, and other experiments.

**Figure 3:**
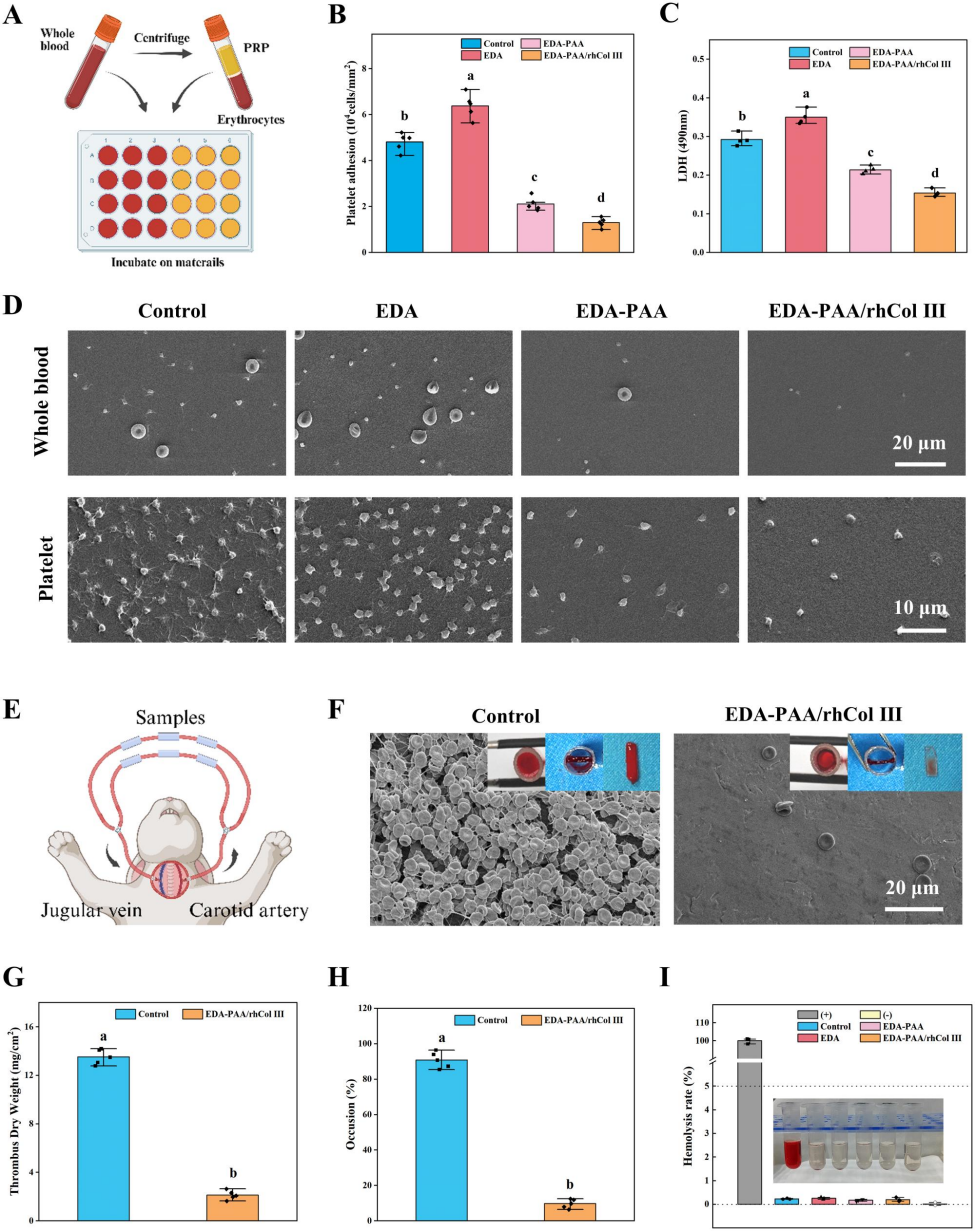
Hemocompatibility evaluation of collagen coating. (**A**) Schematic of static *in vitro* platelet and whole blood adhesion experiments. (**B**) Statistical analysis of platelet adhesion density and (**C**) quantitative analysis of LDH on the sample surfaces. (**D**) SEM images of whole blood and platelet adhesion on the sample surfaces. (**E**) Schematic of rabbit arteriovenous shunt *ex vivo* blood circulation experiment. (**F**) Photographs of the sample surface and catheter cross-section, as well as surface SEM images of different samples, (**G**) thrombus dry weight on the sample surfaces, and (**H**) statistical analysis of the lumen occlusion rate, all after 2 h of *ex vivo* blood circulation. (**I**) Statistical analysis of the hemolysis rates of different samples.

A schematic diagram of the experiments measuring platelet and whole blood adhesion on the sample surface is shown in Figure 3A. Both platelet counting and platelet adhesion assays revealed a significant reduction in platelet adhesion in the coating groups, indicating excellent short-term anticoagulant efficacy of the implanted devices (Figure 3B). This trend was further corroborated by quantitative LDH measurements (Figure 3C). Morphological observations (Figure 3D) showed that numerous RBCs and platelets adhered to the surfaces of the bare substrate and the EDA coating, accompanied by a high level of platelet activation, which is conducive to thrombus formation [63]. In contrast, only a small number of platelets and scattered RBCs were observed on the EDA–PAA and EDA–PAA/rhCol III coating surfaces, validating their superior short-term anticoagulant properties.

*Ex vivo* blood circulation experiments on rabbits were performed to assess antithrombotic characteristics (Figure 3E) [64]. As shown in the SEM images and optical micrographs (Figure 3F), massive thrombi formed by activated platelets, RBCs, and fibrin were observed on the control samples, while only scarce platelet adhesion was detected on the experimental group coated with EDA–PAA/rhCol III.

Statistical analysis of the thrombus dry weight on the material surface and the lumen occlusion rate (Figure 3G and H) revealed that the lumen of the control group was severely occluded, with an occlusion rate of 92.53 ± 4.28%, whereas the thrombus occlusion rate in the EDA–PAA/rhCol III coating group was significantly reduced to only 8.07 ± 1.65%. Moreover, the dry weight of the thrombi in the EDA–PAA/rhCol III-coated group accounted for merely 16.73% ± 2.78% of that found in the control group, signifying that the coating significantly inhibited thrombus formation.

Hemolytic properties are also important indicators for evaluating the hemocompatibility of materials [65]. All samples achieved a hemolysis rate below 1% (Figure 3I), which fulfilled the safety limit of 5% specified in ISO 10993-4 [65]. These results demonstrated that the material and coating posed no hemolytic risk after implantation.

Overall, these findings demonstrated the favorable anticoagulant performance and low hemolytic activity of the EDA–PAA and EDA–PAA/rhCol III coatings, confirming their excellent biocompatibility. The superior biocompatibility of the EDA–PAA coating is mainly attributed to negatively charged carboxyl groups in PAA, which inhibit non-specific blood cell adhesion via electrostatic repulsion, while the high hydrophilicity of PAA forms a dense hydration layer at the blood-material interface to block coagulation-related plasma protein adsorption and suppress endogenous coagulation cascade activation [55, 66, 67]. The anticoagulant efficacy of the coating was further enhanced after rhCol III incorporation. As a core component of native vascular endothelial extracellular matrix (ECM), rhCol III not only retains the anticoagulant effect of the EDA–PAA coating but also inhibits activation of key integrin receptors on platelet membranes, reduces platelet adhesion and aggregation, and suppresses coagulation cascade amplification, thus achieving a more potent and stable anticoagulant effect [32, 68].

### Cytotoxicity testing

Cytotoxicity testing is an important method for evaluating the biocompatibility of medical devices, aiming to detect potential cell damage or toxic reactions caused by materials after contact with human cells. Through cytotoxicity testing, materials can be screened for good biocompatibility, the *in vivo* safety of devices can be predicted, and a scientific basis can be provided for the development and clinical application of devices.

In this experiment, FDA staining marked viable cells, while PI staining indicated dead cells. L929 cells and HUVECs were seeded in 48-well plates at an initial density of 2 × 10^4^ cells per well and then treated with the extracts of nothing, EDA, EDA–PAA and EDA–PAA/rhCol III-coated samples for 24 h, followed by FDA/PI double staining.

**Figure 4:**
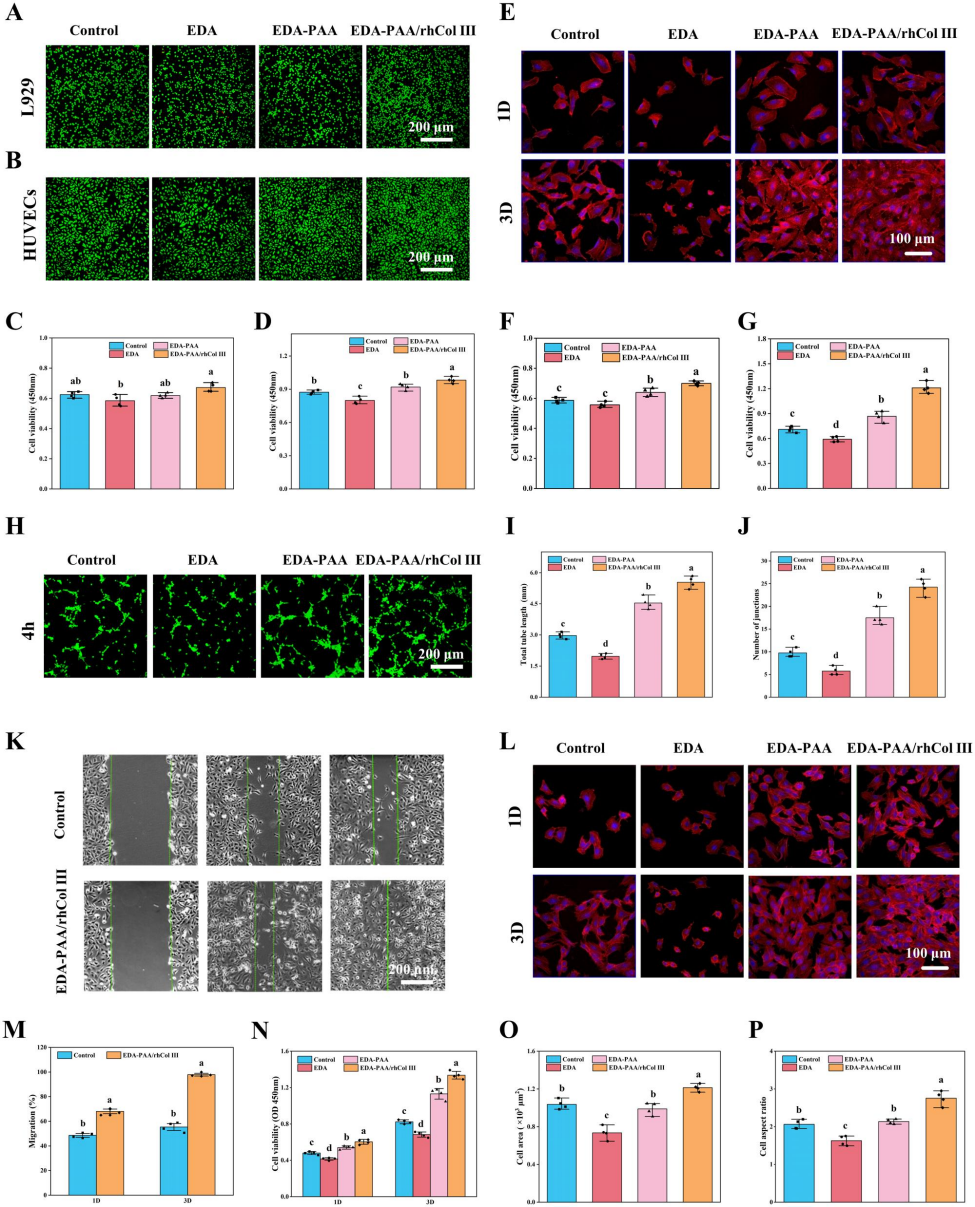
Growth property evaluation of HUVECs and H9c2 cells. (**A** and **B**) FDA/PI double staining images and (**C** and **D**) cell viability of L929 cells and HUVECs after 24 h of culture on the materials. (**E**) CLSM images of HUVEC cytoskeleton staining, and (**F** and **G**) cell viability of HUVECs cultured on the sample surfaces for 1 and 3 days. (**H**) Fluorescence images of HUVECs after 4 h of culture on Matrigel, as well as (**I** and **J**) quantification of total tube length and number of junctions. (**K**) SEM images and (**M**) statistical analysis of the migration rate of H9c2 cells in the wound healing assay after 1 and 2 days of culture on the sample surfaces. (**L**) CLSM images of the cytoskeleton, (**N**) cell viability and pore-averaged quantitative statistics of (**O**) cell area and (**P**) cell aspect ratio of H9c2 cells cultured on the sample surfaces for 1 and 3 days.

Both FDA/PI double staining (Figure 4A and B) and CCK-8 assay (Figure 4C and D) revealed that the EDA coating significantly reduced the viability of L929 cells and HUVECs, accompanied by cell shrinkage and weakened adhesion capacity.

The cytotoxicity of EDA was mainly attributed to its inherent alkalinity. Upon contact with cells, EDA disrupts intracellular pH homeostasis, interferes with normal enzymatic reactions and cellular energy metabolism [69] and triggers excessive accumulation of reactive oxygen species (ROS) [70, 71], which causes oxidative damage to intracellular DNA, proteins and lipids. Meanwhile, the EDA-induced alkaline microenvironment activates intracellular inflammatory signaling cascades, further exacerbating cell damage and inhibiting cell proliferation and adhesion [72, 73]. In contrast, L929 cells and HUVECs cultured on EDA–PAA and EDA–PAA/rhCol III coatings exhibited excellent viability, with normal spreading morphology and no obvious cell death. The complete abrogation of EDA-induced cytotoxicity was ascribed to the synergistic physicochemical neutralization and biological barrier effects of PAA and rhCol III. The grafted PAA intermediate layer achieved core elimination of EDA cytotoxicity through a dual mechanism: on one hand, it neutralized alkaline amino groups of EDA through an acid-base reaction, fundamentally eliminating the cytotoxic root caused by its alkalinity; on the other hand, it formed a dense physical barrier via its hydrated polymer chains to block direct contact between residual EDA and cells, further cutting off the pathway of EDA-mediated cytotoxicity [74]. Furthermore, the covalently grafted rhCol III at the outermost layer of the coating constructed a highly biocompatible and cell-friendly interface, which further offset any trace residual potential cytotoxicity of EDA, forming a final safety guarantee for the biocompatibility of the coating system. These results further confirmed that the EDA–PAA and EDA–PAA/rhCol III coatings were non-cytotoxic to L929 cells and HUVECs, with favorable biocompatibility.

### Growth behavior of HUVECs

To ensure the efficacy of a device, it is crucial to determine whether the device can achieve rapid reendothelialization after implantation—which reduces the risks of thrombus formation and inflammation caused by endothelial damage during implantation and long-term material exposure [51, 75]. In this experiment, the reendothelialization capacity of the EDA–PAA/rhCol III coating was evaluated by assessing the proliferation and migration of HUVECs *in vitro*.

HUVECs were seeded onto the surface of the different materials at a seeding density of 2 × 10^4^ cells/cm^2^, and cell viability and morphology were measured on days 1 and 3. The results (Figure 4E) revealed that the viability of HUVECs was significantly greater on the EDA–PAA/rhCol III coating than on the uncoated surface. As shown in Figure 4F and G, on day 3, the number of HUVECs was reduced in the EDA-coated group, while the number of HUVECs in the EDA–PAA/rhCol III-coated group was significantly increased, with cell viability showing the same trend. The cells were polygonal, with tightly arranged edges and a plumper morphology, indicating a healthy cellular phenotype.

Additionally, angiogenesis is crucial for delivering nutrients and cytokines when tissues undergo repair and regeneration [76]. To clarify the coating’s impact on angiogenesis, we adopted HUVECs to conduct an *in vitro* tube formation assay. As shown in Figure 4H, compared with the control group, the EDA–PAA/rhCol III-coated group formed more tubular networks on the Matrigel. Two primary evaluation criteria for tube formation are total tube length and junction number, with both values increasing significantly among experimental samples (Figure 4I and J). Physiologically, angiogenesis relies on the binding of integrins on vascular endothelial cells to specific domains of collagen, enabling stable adhesion between cells and the ECM [77]. The rhCol III used in this study retains a native triple-helix structure and abundant integrin-binding sites, which facilitate the adhesion, migration and tube formation of HUVECs on Matrigel [60, 78]. The EDA-PAA modified substrate can firmly immobilize rhCol III and preserve its biological activity, consistent with previous findings [79]. These results indicated that the EDA–PAA/rhCol III coating promoted angiogenesis, facilitating the tissue healing process after occluder implantation.

Furthermore, the cell migration results (Figure 4K and M) revealed that under serum-free conditions, 100% migration of HUVECs was achieved on the EDA–PAA/rhCol III coating surface after 2 days, whereas only 56.36 ± 3.92% migration was observed in the control group. As evidenced by the above results, HUVEC adhesion, proliferation and migration were all improved by the EDA–PAA/rhCol III coating, laying a foundation for accelerated endothelialization.

### Growth behavior of H9c2 cells

Notably, the survival and physiological phenotype maintenance of cardiomyocytes are critical for cardiac tissue repair and device integration after LAAO implantation. In this study, H9c2 cells were seeded onto materials with different coatings at a density of 2 × 10^4^ cells/cm^2^ to evaluate the cardiomyocyte compatibility and biological effects of different coatings. The results showed that after 1 and 3 days of culture, the number of H9c2 cells on the EDA–PAA/rhCol III coating was significantly higher than that on the uncoated substrate and EDA-coated surface (Figure 4L), indicating the coating effectively supports cardiomyocyte survival and proliferation with excellent cardiomyocyte compatibility.

This is because the potential cytotoxicity of the EDA coating inhibits cardiomyocyte adhesion and growth, while PAA modification significantly improves the basic biocompatibility of the coating to support normal H9c2 cell growth. RhCol III provides high-affinity adhesion anchor sites for cells via the integrin signaling pathway to enhance adhesion efficiency and promotes the secretion of endogenous collagen and growth-promoting factors to maintain the active proliferative state of cells [80].

Quantitative analysis of the cell area (Figure 4O) and cell aspect ratio (Figure 4P) of H9c2 cells on different coating surfaces revealed that H9c2 cells in the EDA group showed a shrunken spherical morphology, while cells on the EDA–PAA coating had significantly improved spreading area and morphology. Cells in the EDA–PAA/rhCol III group exhibited regular actin arrangement, with a spreading area of 1213.85 ± 37.98 μm^2^ and an aspect ratio of 2.75 ± 0.19. Collectively, compared with the bare, EDA and EDA–PAA groups, the EDA–PAA/rhCol III coating significantly improved cardiomyocyte survival, maintained their physiological functional phenotype, and showed potential to promote tissue repair at the implantation site.

### Inflammatory response

After material implantation, macrophages accumulate on the material and are polarized, resulting in their transformation into two phenotypes: M1 and M2 [81, 82]. M1 macrophages are associated with proinflammatory responses, which further trigger inflammatory reactions, leading to delayed endothelialization and excessive tissue hyperplasia; in contrast, M2 macrophages exert anti-inflammatory effects [83, 84]. The growth behavior of macrophages and the release of inflammatory factors on the material surface are key aspects for evaluating the tissue compatibility of cardiovascular implant materials.

**Figure 5:**
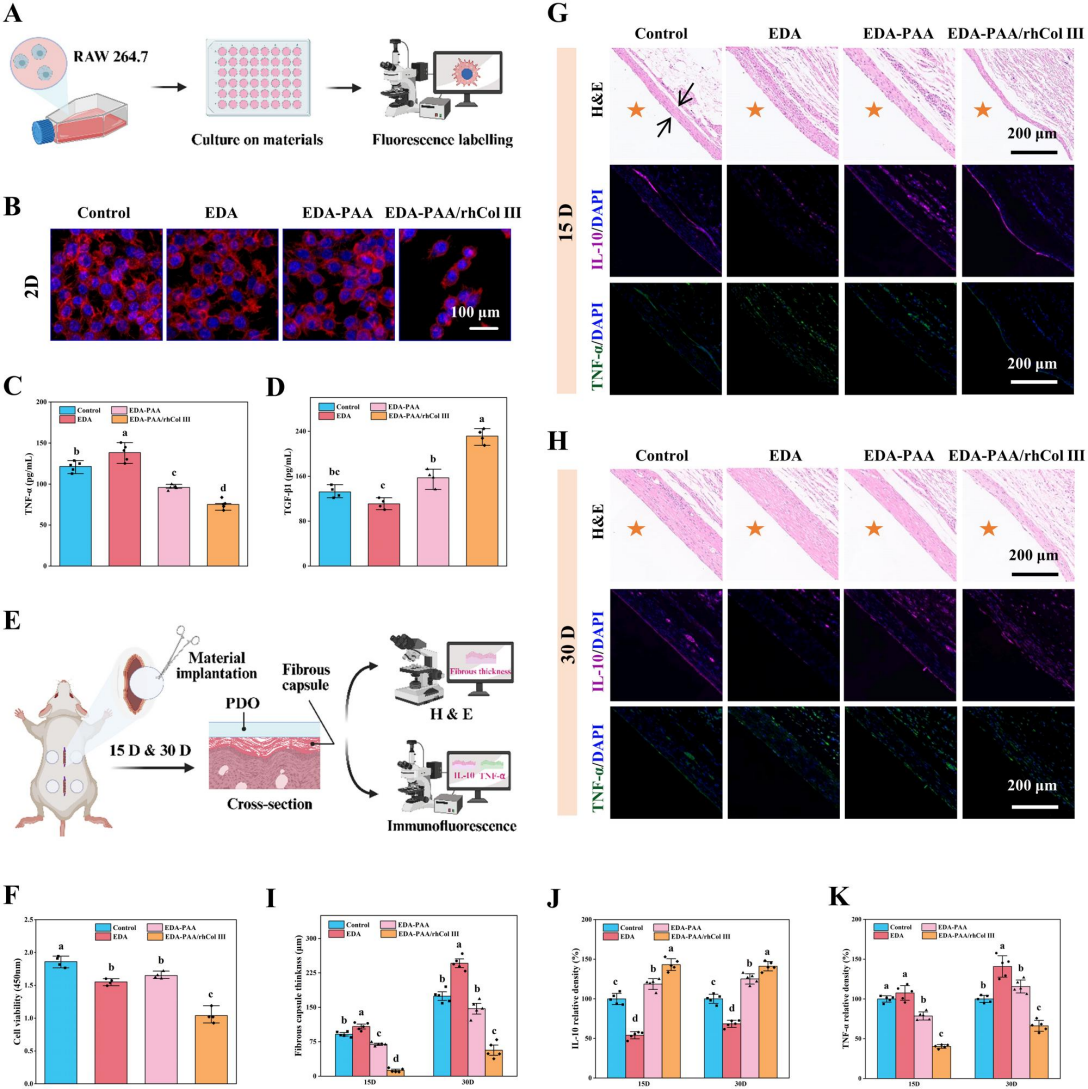
Evaluation of the inflammatory response. (**A**) Schematic diagram of the *in vitro* macrophage experiment. After 2 days of culture on the sample surfaces, (**B**) CLSM images of the cytoskeleton of RAW 264.7 cells, (**C**) quantitative statistical results of TNF-α expression, (**D**) quantitative statistical results of TGF-β1 expression and (**F**) cell viability. (**E**) Schematic diagram of the subcutaneous implantation experiment on the rat dorsum. Hematoxylin-eosin (H&E) and immunofluorescence staining of fibrous capsules encircling different samples following 15 days (**G**) and 30 days (**H**) subcutaneous implantation, along with corresponding quantitative statistical results of (**I**) fibrous capsule thickness, (**J**) IL-10 expression and (**K**) TNF-α expression. The fibrous capsule was labeled by arrows, and the PLA implants marked by orange stars.

RAW 264.7 cells were subjected to enzyme-linked immunosorbent assay (ELISA) detection targeting classically activated macrophages (M1) and alternatively activated macrophages (M2) biomarkers TNF-α and TGF-β1 (Figure 5A). RAW 264.7 macrophages were seeded onto the surface of different coated samples at a seeding density of 4 × 10^4^ cells/cm^2^. Confocal laser scanning microscopy images (Figure 5B) and cell viability data (Figure 5F) of macrophages co-cultured with materials for 2 days showed that macrophages on the control group surface were mostly activated, while those in the EDA-coated group presented an obvious activated phenotype with well-developed pseudopodia. Macrophages on the EDA–PAA coating had significantly improved morphology, appearing as tightly arranged round cells, due to the favorable biocompatibility of PAA supporting normal macrophage proliferation. In contrast, macrophages adhering to the EDA–PAA/rhCol III coating (experimental group) were mostly round or quiescent, with significantly reduced pseudopod structures, indicating that their polarization toward the proinflammatory phenotype (M1 type) was inhibited [85, 86]. ELISA results (Figure 5C and D) revealed that compared with the control group, the EDA–PAA group showed lower TNF-α and higher TGF-β1 expression, while rhCol III incorporation further reduced TNF-α and significantly upregulated TGF-β1 expression.

These results indicated that the EDA–PAA/rhCol III coating surface could inhibit macrophage adhesion, regulate cell polarization, and reduce the expression of proinflammatory factors, thereby alleviating inflammatory responses.

Subcutaneous implantation is a commonly used approach for investigating inflammatory responses at the material implantation site [51]. The *in vivo* inflammatory response to the EDA–PAA/rhCol III coating was evaluated by implanting PDO sheets into the subcutaneous tissue of the rat dorsum (Figure 5E). After material implantation, a positive correlation exists between fibrous capsule thickness and the degree of local inflammatory response in this model [87]. As shown in Figure 5G and H, compared with that of the control group (176.67 ± 18.29 μm), the fibrous capsule thickness of the EDA group (233.10 ± 40.05 μm) increased 30 days after implantation. In contrast, the fibrous capsule thickness in the EDA–PAA group (144.59 ± 23.58 μm) and EDA–PAA/rhCol III coating group (55.67 ± 14.05 μm) significantly decreased, which was consistent with the results after 15 days of implantation.

Additionally, immunofluorescence analysis (Figure 5J and K) revealed that, compared with the control group, distinct fluorescence signals of interleukin-10 (IL-10) were observed in the outer layer of the fibrous capsule in both the EDA–PAA and EDA–PAA/rhCol III coated groups at 15 days and 30 days post-implantation, with more continuous signals detected in the EDA–PAA/rhCol III coated group. Meanwhile, the fluorescence signal of TNF-α was significantly reduced in the EDA–PAA/rhCol III-coated group. These findings indicated that the EDA–PAA/rhCol III-coated group had a higher expression level of the anti-inflammatory factor IL-10, and the inflammatory response triggered by the EDA–PAA/rhCol III-coated material at the material–blood interface was alleviated.

These results demonstrated that rhCol III realized the synergistic amplification of anti-inflammatory and pro-reparative effects based on the EDA–PAA coating, and the EDA–PAA/rhCol III coating could regulate inflammatory responses by promoting macrophage polarization toward the anti-inflammatory M2 phenotype, thereby improving the material’s tissue compatibility.

### Intravascular PDO monofilament implantation

**Figure 6:**
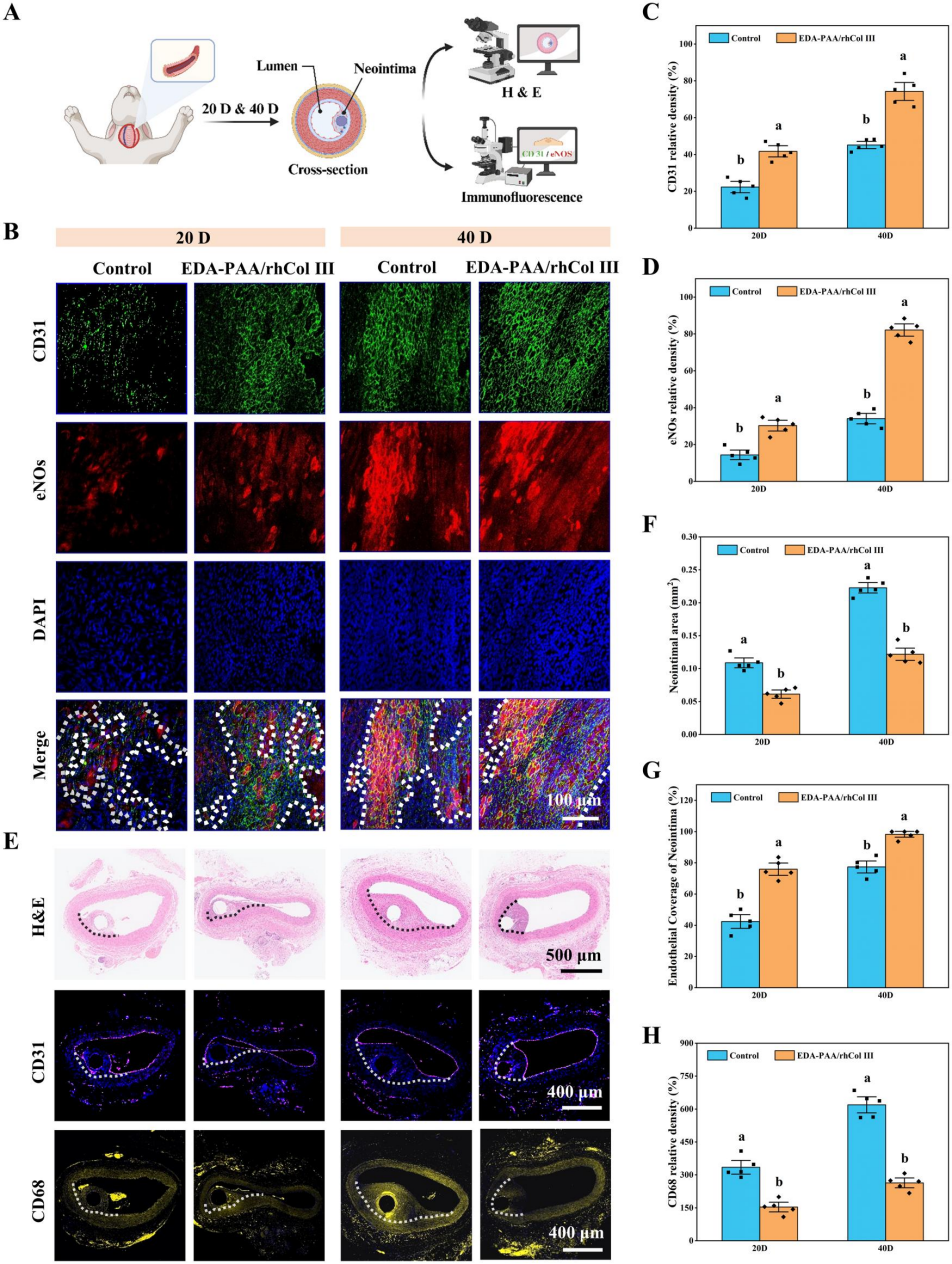
Evaluation of endothelial regeneration and inflammation in intravascular PDO monofilament implants. (**A**) Schematic diagram of the PDO monofilament implantation experiment in rabbit carotid arteries. (**B**) Immunofluorescence images of CD31 and eNOS expression on the surfaces of bare and EDA–PAA/rhCol III-coated PDO monofilaments 20 and 40 days after implantation, along with (**C** and **D**) quantitative statistical data. Non-endothelialized areas are circled with white dashed circles. (**E**) H&E staining and immunohistochemical images of vascular cross-sections containing monofilaments 20 and 40 days after implantation, as well as corresponding quantitative statistical data on (**F**) neointimal area, (**G**) CD31 expression and (**H**) CD68 expression. Dashed lines indicate the boundary between the native blood vessel and the neointima.

After LAAO implantation into the heart, rapid surface reendothelialization and internal tissue regeneration are required to occlude the defect area. PDO monofilaments—the scaffold material of occluders with coatings—were implanted into the carotid arteries of rabbits (Figure 6A) to evaluate the *in vivo* reendothelialization and tissue healing effects of the EDA–PAA/rhCol III coating.

Immunofluorescence analysis was used to measure the expression of platelet–endothelial cell adhesion molecule (PECAM-1/CD31) and eNOS on the surface of the PDO monofilaments to assess the reendothelialization of the coating. CD31, a canonical endothelial marker enriched at inter–endothelial tight junctions, reflects endothelial monolayer integrity [32, 88], while eNOS—the key rate-limiting enzyme for NO synthesis in mature endothelial cells—indicates endothelial functional maturity via NO-mediated vascular homeostasis regulation [25, 89]. High expression of CD31 and eNOS further verifies the structural integrity and biological function of the nascent endothelial layer.

As illustrated in Figure 6B–D and Supplementary material S3, sparse and fragmented CD31 and eNOS expression was detected on the pristine PDO surface at 20 days post-implantation, with respective levels of 20.31 ± 6.10% and 11.85 ± 2.74%. Compared with those in the bare PDO group, the expression densities of CD31 (40.74 ± 5.42%) and eNOS (32.30 ± 1.19%) in the EDA–PAA/rhCol III coating group significantly increased. At 40 days after implantation, the expression of CD31 and eNOS in both groups increased to varying degrees, whereas the EDA–PAA/rhCol III coating group exhibited a more intact, uninterrupted and abundant distribution of CD31 and eNOS on the surface.

By calculating the coverage area of regularly growing newly formed endothelial cells on the basis of the distribution of CD31 and eNOS, it was found that 40 days after implantation, the vascular endothelialization coverage rate reached 71.68 ± 5.13%, which was significantly greater than the 39.10 ± 2.54% of the control group. These outcomes corresponded to the *in vitro* growth performance of HUVECs. The EDA–PAA/rhCol III coating facilitated accelerated and fuller endothelial cell coverage, indicating that the EDA–PAA/rhCol III coating accelerated the reendothelialization process after occluder implantation.

Inflammatory responses after LAAO implantation may cause tissue erosion and rhythm disturbance while impeding reendothelialization and tissue healing. During intravascular implantation, persistent inflammation can also stimulate excessive neointimal hyperplasia, and neointimal thickness is positively correlated with inflammatory progression [87]. Staining of vascular cross-sectional tissues via H&E (Figure 6E and F) revealed that 20 days after implantation, the neointimal area of the EDA–PAA/rhCol III-coated group (0.06 ± 0.01 mm^2^) was significantly smaller than that of the bare PDO group (0.11 ± 0.02 mm^2^). At 40 days after implantation, the neointimal area of the bare PDO group increased to 0.22 ± 0.04 mm^2^, whereas that of the EDA–PAA/rhCol III group was only 0.12 ± 0.01 mm^2^, which was much lower than that of the bare PDO group. These results indicated that the EDA–PAA/rhCol III coating minimally stimulated intimal hyperplasia and resulted in a mild inflammatory response.

As a typical macrophage biomarker, CD68 can partly evaluate inflammatory severity, and its elevated expression is accompanied by increased infiltration of inflammatory cells. CD68 immunohistochemical staining was performed on vascular cross-sections, as displayed in Figure 6G and H. The results showed that specimens modified with EDA–PAA/rhCol III had a lower CD68-positive density than untreated PDO samples after implantation. Highly consistent with H&E histological observations, these experimental data proved that the EDA–PAA/rhCol III coating is capable of effectively mitigating inflammatory reactions.

In conclusion, the EDA–PAA/rhCol III coating fabricated onto PDO monofilaments was verified to enhance reendothelialization at the material surface, alleviate inflammation and facilitate rapid tissue healing at the implantation site. Owing to its excellent tissue compatibility, rhCol III improved the surface hemocompatibility of the material to reduce the nonspecific adsorption of plasma proteins, inhibit platelet adhesion and activation, and simultaneously reduce the release of local inflammatory factors by suppressing the polarization of macrophages toward the proinflammatory M1 phenotype. Additionally, it promoted the proliferation, migration, and functional expression of HUVECs. Thus, EDA–PAA/rhCol III coating is highly promising for serving as a surface modification material of LAAOs.

Nevertheless, the rabbit carotid artery implantation model used cannot fully replicate the complex anatomy, hemodynamics, and contractile microenvironment of the human LAA, particularly under AF. To contextualize the rationality of this *in vivo* model, we first confirmed its core similarities to the clinical LAAC setting. The PDO implant matched the mainstream scaffold material of clinical LAAC devices with consistent degradation kinetics and surface physicochemical properties. Both involved long-term implant contact with circulating blood to evaluate the coating’s core functions of hemocompatibility, endothelialization and inflammatory modulation. And both recapitulated the implant–host endothelial interface interaction to provide robust proof-of-concept data [15, 20].

Meanwhile, key discrepancies exist between this model and clinical LAAC. Hemodynamically, the LAA (especially under fibrillation) features a low-shear, low-velocity, stagnant flow environment with bidirectional cyclic flow [7, 90]. In contrast, the rabbit carotid artery presents unidirectional, medium-to-high velocity laminar flow with significantly higher shear stress, which directly influences platelet activation, thrombus formation and endothelial phenotype [20]. Anatomically, the LAA has a trabeculated, irregular surface lined with phenotypically distinct atrial endocardium, unlike the smooth, continuous vascular lumen of the carotid artery. Furthermore, clinical LAAC devices are three-dimensional mesh scaffolds exerting radial support and multi-point endocardial contact, which cannot be fully recapitulated by the linear monofilament implant in this model.

Therefore, a primary focus of our future work will be to fabricate this coating on full-scale occluders and systematically evaluate its structural integrity after simulated deployment, its antithrombotic performance under conditions that more closely resemble physiological geometry and hemodynamics, and its long-term (such as >6 months) implantation safety and efficacy in large animal (such as porcine) models of cardiac defects.

### Transcriptomic analysis reveals immune regulation and *in situ* endothelialization during endogenous tissue regeneration

To elucidate the molecular mechanisms underlying the pro-endothelialization effect of the EDA–PAA/rhCol III coating, we performed transcriptomic analysis on *in vitro* cultured HUVECs grown on different material surfaces for 72 h. We focused on gene expression profiles associated with core cellular processes mediating *in situ* endothelialization (cell adhesion, migration, proliferation, anti-apoptosis) and immune microenvironment regulation.

**Figure 7:**
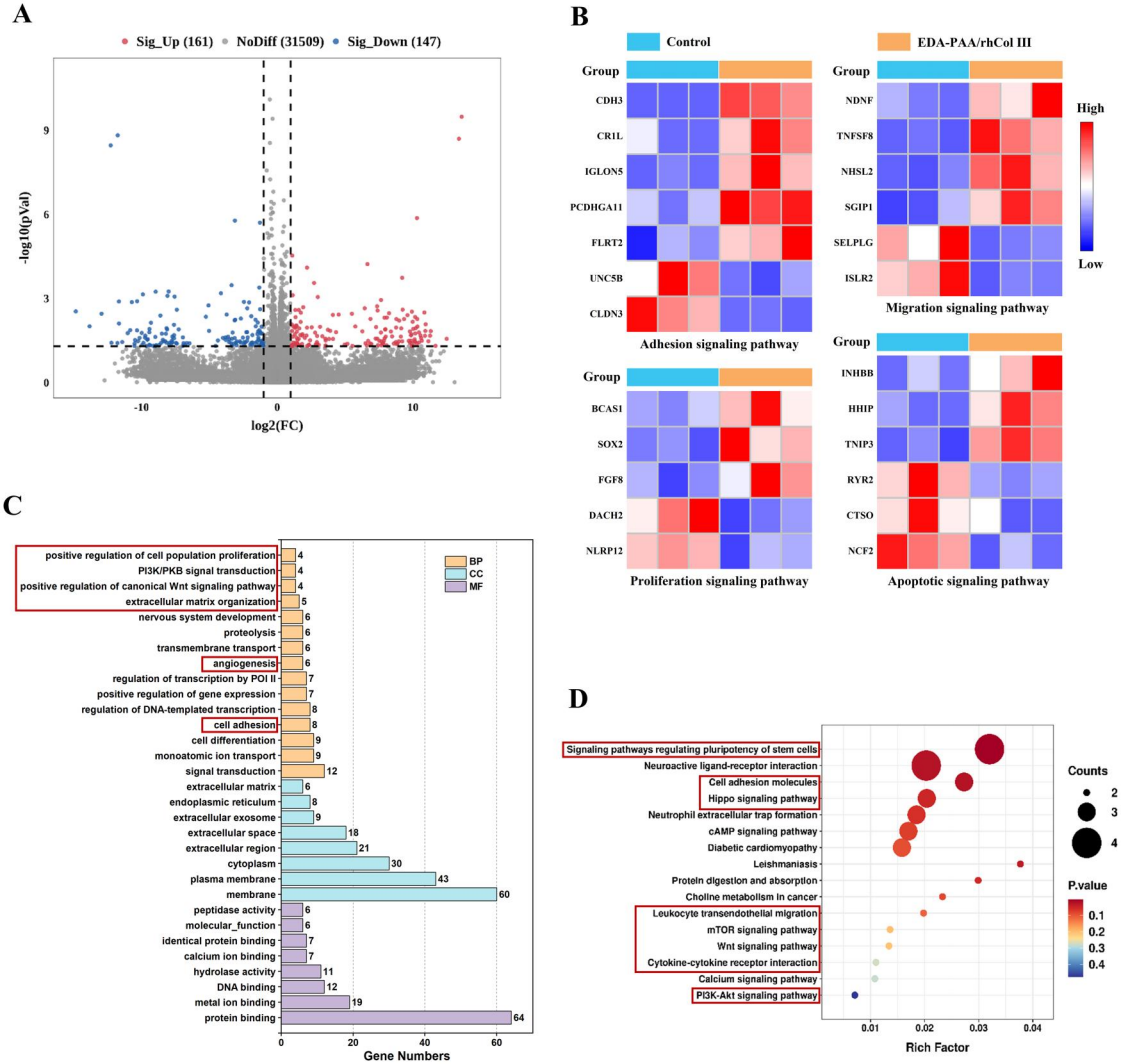
Transcriptomics reveals immune regulation and endothelialization processes. (**A**) Volcano plot and (**B**) clustered heatmap of significantly upregulated and downregulated differentially expressed genes (DEGs) identified by transcriptomic analysis. The highlighted data points represent DEGs selected on the basis of an adjusted *P-*value < 0.05 and an absolute value of log_2_ fold change (log_2_FC) > 1. (**C**) GO pathway enrichment analysis of the DEGs. (**D**) KEGG pathway enrichment analysis of the DEGs. BP, biological process; CC, cellular component; MF, molecular function.

A total of 308 DEGs were identified between the EDA–PAA/rhCol III coating group and the bare PDO control group, including 161 upregulated and 147 downregulated genes (Figure 7A). Hierarchical clustering heatmap of DEGs showed distinct transcriptomic profiles between the two groups (Figure 7B, full gene names and abbreviations listed in Supplementary Table S2).

GO and KEGG enrichment analyses were performed to identify core biological processes and signaling pathways modulated by the rhCol III–functionalized coating (Figure 7C and D). GO enrichment analysis revealed that the most significantly enriched biological processes included positive regulation of endothelial cell proliferation, angiogenesis, cell–matrix adhesion and ECM organization; molecular function terms were predominantly enriched in protein binding and integrin binding activity. KEGG pathway enrichment further identified four core regulatory pathways: CAMs, PI3K–Akt signaling pathway, Hippo signaling pathway and neutrophil extracellular trap formation (Figure 7D). These enriched pathways directly corresponded to the pro-endothelialization, anti-inflammatory and pro-healing phenotypes observed in our *in vitro* and *in vivo* experiments.

We next characterized key DEGs enriched in the two core biological themes of the study: *in situ* endothelialization and immune regulation. For endothelialization-related processes, we identified significant upregulation of cell adhesion-associated genes (*FLRT2* [74], *CDH3* [26], *CR1L* [91]), which are critical for maintaining endothelial monolayer integrity and promoting cell–cell/cell–matrix interactions during tissue regeneration. Meanwhile, downregulation of *UNC5B* [92] relieved its inhibitory effect on the Notch signaling pathway, further enhancing endothelial cell proliferation and migration. Upregulation of migration-promoting genes (*NDNF* [93], *SYT2* [94]) facilitated directional migration and recruitment of endothelial cells to the coating surface for tissue repair [95]. For cell survival and proliferation, upregulation of *HHIP* [96] and *FGF8* [97] inhibited endothelial apoptosis via activation of the PI3K–Akt pathway, while *FGF8* also stimulated endothelial cell proliferation by activating downstream MAPK signaling via FGFR ligation. In contrast, downregulation of pro-apoptotic genes (*NCF2* [98], *RYR2* [99]) reduced inflammatory factor secretion and subsequent endothelial apoptosis.

Based on the key DEGs identified from our transcriptomic analysis, we considered that the EDA–PAA/rhCol III coating modulated the biological behaviors of HUVECs primarily via the PI3K/Akt/mTOR signaling cascade. The residual carboxyl groups in the EDA–PAA coating backbone possess excellent cytocompatibility [100]. The core bioactive component rhCol III binds to integrin receptors on the endothelial cell membrane via its intrinsic integrin–binding domains [26, 32]. Our transcriptomic data revealed that the EDA–PAA/rhCol III coating significantly upregulated *SOX2* expression in HUVECs. *SOX2* upregulates *PIK3CA* transcription by recruiting KLF4 to its promoter region, triggering the downstream PI3K/Akt cascade [101]. Concurrently, rhCol III-induced *FGF8* upregulation directly activates PI3K and subsequent Akt phosphorylation via binding to the FGFR tyrosine kinase receptor [102]. Activated phospho-Akt senses growth factor, bioenergetic, cellular stress, and amino acid signals and drives HUVEC growth and proliferation through promoting anabolic programs including transcription, translation, and lipogenesis [103]. Furthermore, *CLDN3* downregulation also regulates endothelial adhesion and migration via modulating PI3K/Akt activation [104].

For immune regulation and anti-inflammatory processes, activation of the PI3K/Akt signaling axis further inhibits the nuclear translocation of NF-κB, thereby suppressing the transcription and secretion of pro-inflammatory cytokines including TNF-α, IL-6 and IL-1β in macrophages and vascular endothelial cells [105]. We identified significant downregulation of multiple pro-inflammatory genes. *SELPLG* [106] downregulation suppressed vascular inflammation via inhibition of the NF–κB pathway, while also relieving functional inhibition of endothelial progenitor cells to promote angiogenesis. Downregulation of *NLRP12* [107] and *NCF2* alleviated endothelial inflammatory responses and inhibited excessive neointimal hyperplasia, which in turn created a favorable microenvironment for rapid endothelialization.

Collectively, these pathways crosstalked to form an integrated regulatory network governing endothelial adhesion, proliferation, migration, metabolism and angiogenesis [108], which coordinately maintained the homeostasis of the cellular microenvironment and facilitated vascular regeneration.

These molecular mechanisms were fully consistent with the pro-endothelialization, anti-inflammatory, and pro-healing phenotypes observed in our *in vitro* and *in vivo* experiments. On this basis, it was necessary to compare our coating with commercial LAAOs to clarify its clinical translation potential. Internationally, the commercially dominant WATCHMAN FLX Pro occluder uses a specific antithrombotic physically deposited PVDF-HFP coating [16, 109], which enhances antithrombotic effects by precisely controlling the coating thickness and surface roughness and improves coating durability via advanced interface bonding technology. Domestically, research and development centers on the localized innovation of coating materials. For instance, the LAmax LAAO adopts the SMART negative ionization-modified PET membrane technology, which introduces negative charges onto the surface of the PET membrane, thereby reducing platelet adhesion and enhancing surface hydrophilicity and biocompatibility [110]. Despite their widespread clinical application, these occluders still lack the capacity for active anticoagulation, anti-inflammation, and tissue regeneration.

In contrast to these existing strategies, our EDA–PAA/rhCol III coating achieved stable covalent immobilization via a three-tier amide bond network, integrated anticoagulant, anti-inflammatory, and pro-endothelialization functions, and realized a nearly 2-fold higher endothelialization rate at 40 days post-implantation. We further compared the core performance indicators (anticoagulant effect, anti-inflammatory effect, endothelialization efficiency and coating stability) of our coating-modified LAAC device with four clinically approved commercial products, and the results showed that our coating exhibited significant advantages in all the above indicators (Supplementary Table S3) [111, 112].

### Limitations and future perspectives

Despite promising findings, several limitations should be acknowledged. First, while our *in vitro* system systematically characterized coatings with different component combinations, *in vivo* experiments only verified the efficacy and biosafety of the final optimized EDA–PAA/rhCol III coating. No parallel *in vivo* comparisons of single and dual-component materials were performed, preventing direct quantification of each component’s independent contributions and synergistic mechanisms.

Furthermore, while rhCol III enhanced endothelialization and anti-inflammatory activity on the EDA–PAA base, its specific advantages over other natural proteins and synthetic coatings in this system have not been fully established.

## Conclusions

Transcatheter LAA closure has become a mainstream therapy for stroke prevention in patients with AF, yet its long-term clinical efficacy remains persistently limited by post-implant DRT, delayed reendothelialization, and chronic inflammation. To address these unmet clinical needs, we developed a hierarchical multifunctional coating via stepwise covalent grafting of EDA, PAA and rhCol III onto biodegradable polymer substrates for LAAC device surface modification.

The three-step covalent grafting strategy developed in this study constructs a stable three-tier amide bond network within the coating, which effectively overcomes the critical challenge of *in vivo* exfoliation that plagues conventional collagen coatings. The rationally designed EDA–PAA interlayer not only eliminates the intrinsic cytotoxicity of EDA via acid-base neutralization but also endows the coating with natural anticoagulant properties. The introduction of rhCol III further incorporates multiple bioactivities including pro-endothelialization, immunomodulation and cardiomyocyte function maintenance, leading to a substantial improvement in the biocompatibility of the material. This hierarchical design allows the EDA–PAA/rhCol III coating to integrate the three core functions of anticoagulation, anti-inflammation and pro-endothelialization, thereby resolving the long-standing limitations of monofunctionality and insufficient tissue repair efficacy in existing coatings for cardiovascular implantable devices and providing a novel theoretical and technical solution for the surface modification of cardiovascular implantable devices.

Systematic *in vitro* and *in vivo* evaluations demonstrated that the EDA–PAA/rhCol III coating exhibits excellent physicochemical stability, hemocompatibility, and cytocompatibility. It effectively inhibits platelet adhesion and thrombus formation, promotes endothelial cell proliferation, migration and angiogenesis, optimizes cardiomyocyte phenotypic function and mitigates inflammatory responses by driving macrophage polarization toward the anti-inflammatory M2 phenotype. Notably, the coating achieved 71.68% endothelialization coverage 40 days post-implantation in a rabbit carotid artery model, nearly double that of the unmodified control group. Transcriptomic analysis further revealed that its pro-regenerative and immunomodulatory effects are mainly mediated by PI3K-Akt and Hippo pathway activation. Compared with globally mainstream commercial LAAO systems including WATCHMAN FLX Pro, Lambre, Amplatzer Amulet and LAMax devices, our EDA–PAA/rhCol III-coated device presents prominent advantages in active endothelial repair, long-term *in vivo* structural stability, and scalable potential for clinical and industrial translation.

This study offers a robust, clinically translatable technical solution to drive the paradigm shift of LAAC therapy from conventional mechanical occlusion to biofunctional *in situ* vascular regeneration.

## Supplementary Material

rbag114_Supplementary_Data

## Data Availability

All raw experimental data generated and analyzed during this study are available from the corresponding author upon reasonable request.
