# Supplementary material for: A recombinant humanized type III collagen coating with anti-inflammatory and endothelialization-promoting effects for left atrial appendage closure devices
Source: Regen Biomater. 2026 Jun 5;13:rbag114. doi: 10.1093/rb/rbag114 (PMC13332433; doi:10.1093/rb/rbag114)
Supplement: rbag114_Supplementary_Data [file rbag114_supplementary_data.docx]

**Supporting information**

**A Recombinant Humanized Type III Collagen Coating with Anti-Inflammatory and Endothelialization-Promoting Effects for Left Atrial Appendage Closure Devices**

Yihang Zhou^a^, Jiaqi Wang^a^, Longjian Zhang^a^, Li Yang^*,a^, Yunbing Wang^a,b,c^

^a^ National Engineering Research Center for Biomaterials and College of Biomedical

Engineering, Sichuan University; Chengdu, 610065, China

^b^ Research Unit of Minimally Invasive Treatment of Structural Heart Disease, Chinese Academy of Medical Sciences, Beijing, 100730, China

^c^ Chengdu Minshan Institute of Biomaterials, Chengdu, 610200, China

^*^: Corresponding author

Email: [yanglisc@scu.edu.cn](mailto:yanglisc@scu.edu.cn)

This file contains:

- Supplementary Text M1 to M6
- Fig. S1 to S3
- Table S1 to S3

**Supplementary Text**

**M1.** Preparation of FITC-conjugated rhCol III

For fluorescein isothiocyanate (FITC)-conjugated rhCol III, 2 mg/mL rhCol III solution prepared with sodium carbonate buffer (0.1 M, pH 9.0) and 1 mg/mL FITC solution prepared with dimethyl sulfoxide were mixed with a volume ratio of 20:1, and the mixed solution was stirred at 25°C for 18 h. Subsequently, ammonium chloride solution (50 mM) was added and stirred at 25°C for 2 h to terminate the reaction. Next, the mixed solution was dialyzed against ultrapure water with a dialysis bag (MWCO: 3500 Da) for 3 days and lyophilized to obtain FITC-conjugated rhCol III. All experimental operations were carried out under dark conditions.

**M2.** LDH test

Platelet adhesion was quantitatively analyzed by a lactate dehydrogenase (LDH) cytotoxicity test kit. First, the lactic acid solution, iodonitrotetrazolium chloride (INT) solution (diluted 10 times with PBS), and enzyme solution in the kit were mixed with a volume ratio of 1:1:1 to prepare the LDH detection working solution. The samples were co-incubated with 300 μL of PRP at 37°C for 1 h and rinsed 3 times with PBS. Subsequently, they were co-incubated with 300 μL of LDH releasing reagent (diluted 10 times with PBS) at 37°C for 1 h. Afterwards, 120 μL of supernatant from each group was mixed with 60 μL of LDH detection working solution, and shaken at 75 rpm in a dark environment at 25°C for 30 min. Finally, the absorbance of the mixed solution was detected at 490 nm using a microplate reader.

**M3.** The Cytotoxicity test of L929 cells and HUVECs

The sterilized PDO, EDA, EDA–PAA, and EDA–PAA/rhCol III-coated PDO sheets (1 cm × 1 cm) were were incubated with L929 and HUVEC suspension at a density of 2 × 10^4^ cells/mL for 1 and 3 days. Adherent L929 cells and HUVECs on the material surface were observed using an inverted fluorescence microscope after after being co-stained with fluorescein diacetate (FDA) and propidium iodide (PI).

**M4.** Survival, proliferation, migration, and tube formation of HUVECs

The sterilized PDO sheets were incubated with HUVEC suspension at a density of 2 × 10^4^ cells/mL for 1 and 3 days. Adherent HUVECs on the material surface were observed using an inverted fluorescence microscope, and cell viability was evaluated using the CCK-8 assay. Moreover, the intact cell layer on the sample surface was scratched with a sterile 1 mL pipette tip along a straight line to form gaps approximately 300 μm wide and cultured with FBS-free medium for 1 and 2 days. Subsequently, HUVEC migration was observed using an inverted fluorescence microscope, and the migration rate was calculated by the ratio of the sum of the migration distances on both sides to the initial gap distance (300 μm).

Furthermore, different samples were immersed in the medium at 37°C for 1 day to prepare extracts. The HUVEC suspension at a density of 4 × 10^4^ cells/mL and sample extracts (v/v, 1/1) were added to the solidified Matrigel (BD Biocoat 356231, Corning, NY, USA) surface and incubated for 8 h. After FDA staining, tube formation was observed using an inverted fluorescence microscope, and the number of junctions and total tube lengths were analyzed using ImageJ software with the specialized AngioTool plugin.

**M5.** Proliferation and morphology of H9C2 cells

The proliferation of H9C2 cells were evaluated by the CCK-8 assay in the same manner as for HUVECs. Cells cultured on different sample surfaces for 1 day and 3 days were sequentially stained with tetramethylrhodamine isothiocyanate (TRITC)-conjugated phalloidin (Solarbio, Beijing, China) and 4’,6-diamidino-2-phenylindole (DAPI, Solarbio, Beijing, China). The cytoskeleton was observed using confocal laser scanning microscopy (CLSM), and the area and aspect ratio of individual H9C2 cells were calculated using ImageJ software (n = 25). Specifically, a total of 25 images of cytoskeleton staining were collected from 5 parallel samples in each group. The area and aspect ratio of all cells in each image were calculated by the ImageJ software, and the average value was taken as an independent sample value. The final quantitative results were obtained by statistical analysis of the average values from 25 images.

**M6.** The proliferation ability, morphological characteristics, and TNF-α and TGF-β expressions in RAW264.7 cells

The survival and proliferation of RAW264.7 cells were evaluated by the CCK-8 assay in the same manner as for HUVECs. The RAW264.7 Cells cultured on different sample surfaces for 2 days were sequentially stained with tetramethylrhodamine isothiocyanate (TRITC)-conjugated phalloidin (Solarbio, Beijing, China) and 4’,6-diamidino-2-phenylindole (DAPI, Solarbio, Beijing, China). The cytoskeleton was observed using confocal laser scanning microscopy (CLSM).

The expressions of macrophage phenotypic markers TNF-α and TGF-β in RAW264.7 cells were evaluated via ELISA Kit for Transforming Gronth Factor Beta 1 (TGFb1) and ELISA Kit for Tumor Necrosis Alpha (TNFα) (Cloud-Clone Corp., USA). Fluorescence images were captured using CLSM, and the relative densities of TNF-α and TGF-β were calculated using ImageJ software based on the fluorescence intensity (set the control to 100%).

**Fig. S1.** Surface element composition ratio of bare, EDA, EDA-PAA, and EDA-PAA/rhCol III coated (A) PLA and (B) PDO substrates by XPS. Relative proportions of each component derived from peak fitting analysis of the coatings on the PLA (C) and PDO (D) substrates


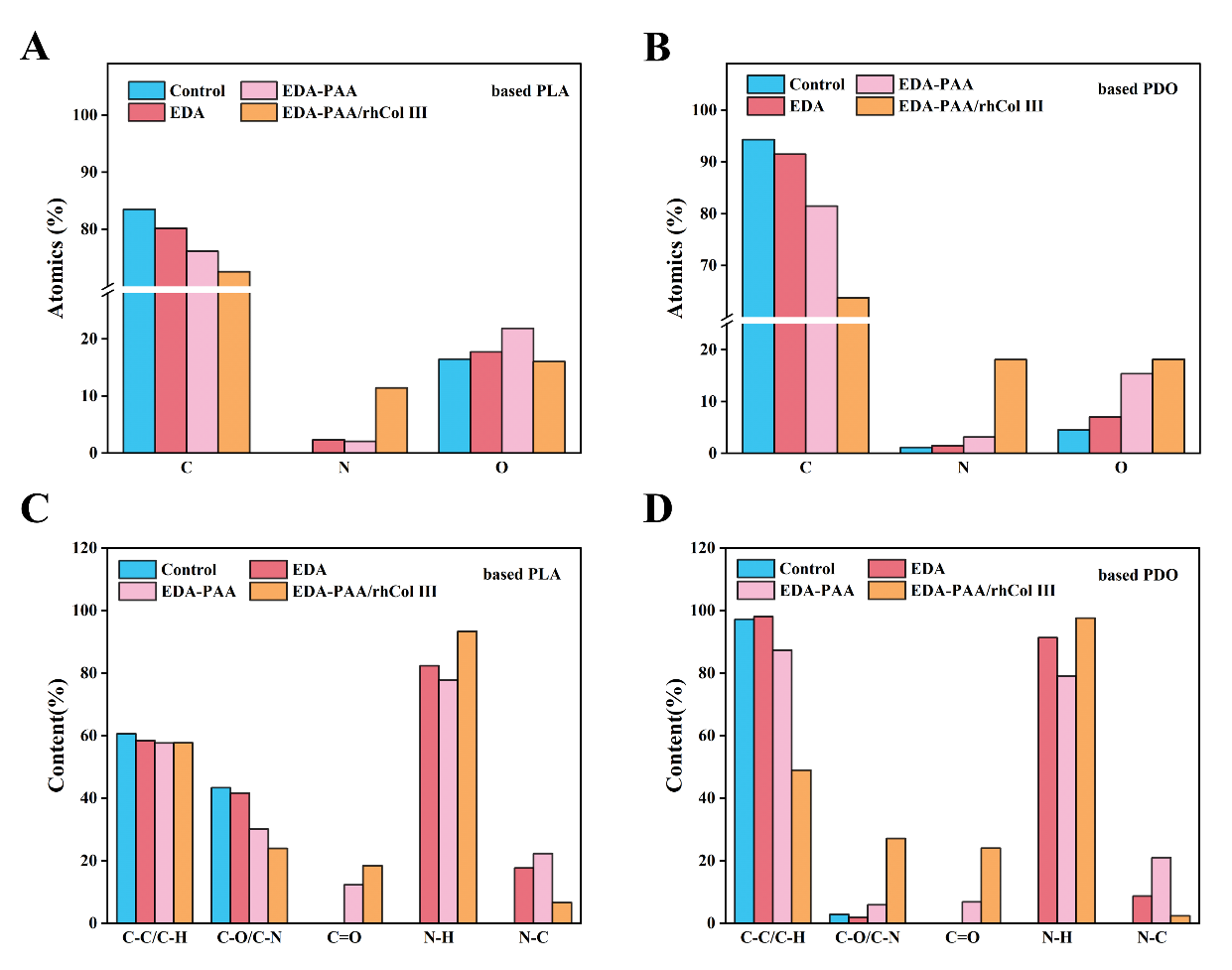


**Fig. S2.** XPS survey scan spectra, and surface elemental composition ratio of the bare, EDA, EDA-PAA and EDA-PAA/rhCol III coated PLA substrates after immersion in PBS for 0, 7, and 14 days.


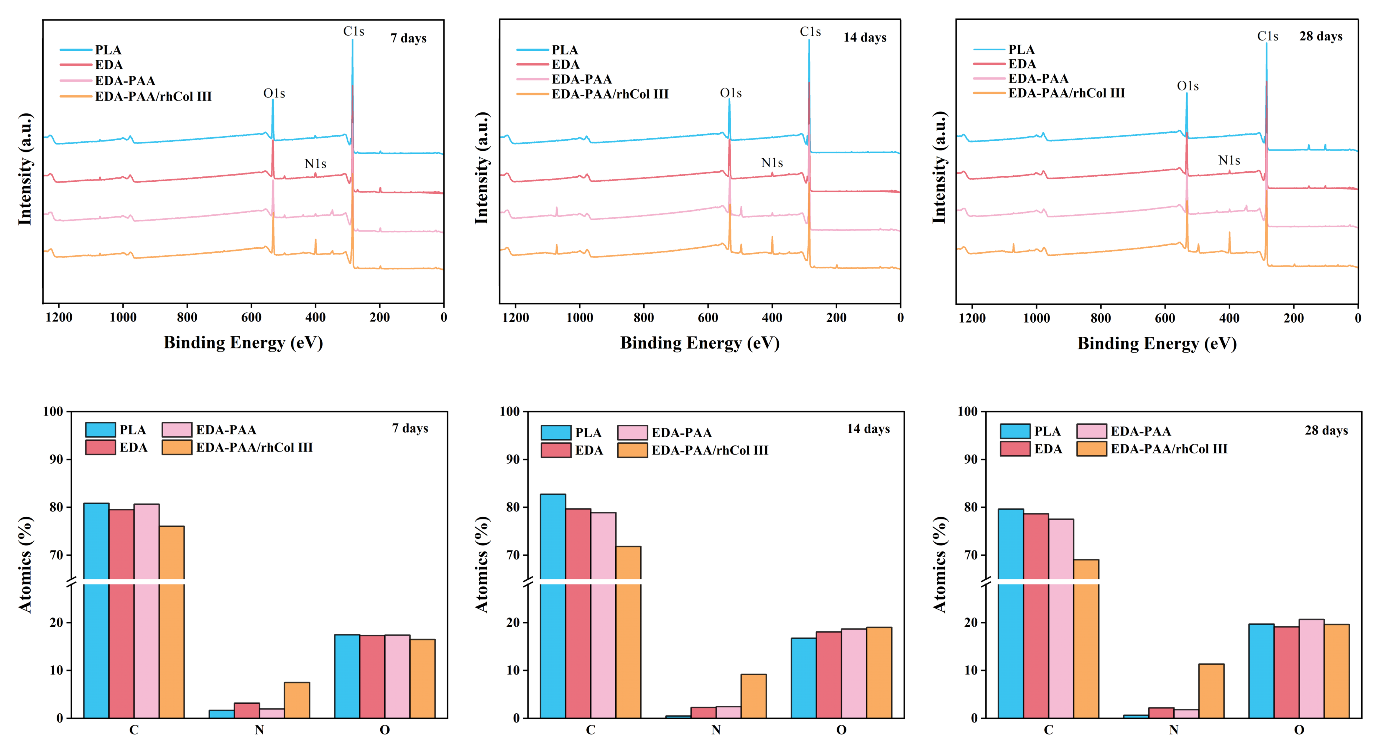


**Fig. S3.** Immunofluorescence images of CD31 and eNOS expression on the inner wall of the healthy rabbit’s carotid artery.


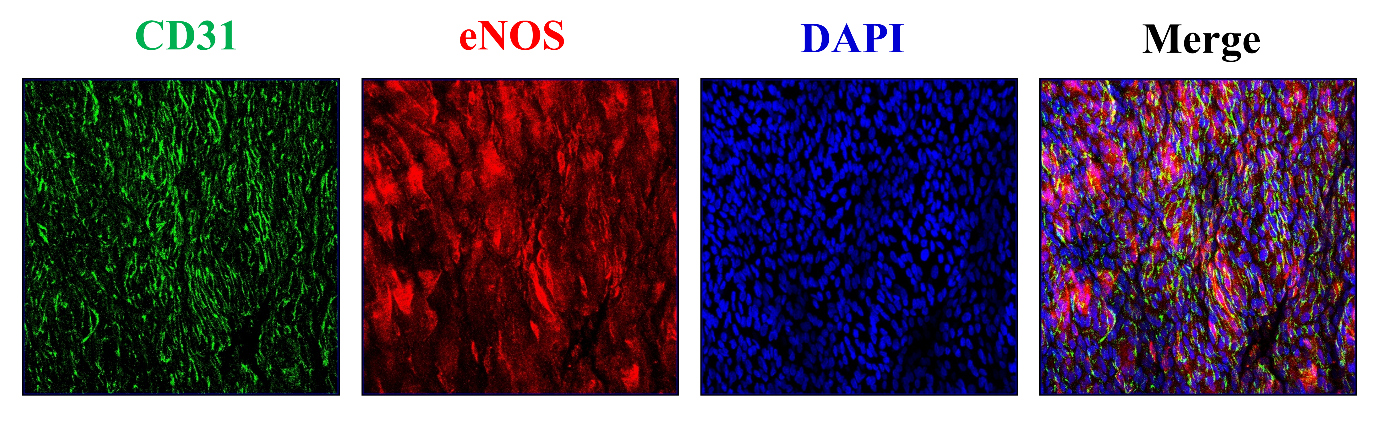


**Table S1.** The proportions of different elements obtained by Energy Dispersive Spectrometer（EDS) on bare, EDA, EDA-PAA and EDA-PAA/rhCol III coated PLA substrates.

|  | **Atomic (%)** | | | |
| --- | --- | --- | --- | --- |
| Element | Control | EDA | EDA–PAA | EDA–PAA/rh Col III |
| C | 79.70 ± 1.95% | 80.36 ± 1.76% | 80.61 ± 2.36% | 81.87 ± 1.44% |
| O | 20.31 ± 1.05% | 19.64 ± 0.84% | 19.39 ± 0.71% | 18.13 ± 0.39% |

**Table S2.** Abbreviations and full names of genes labeled in the clustered heatmap of HUVECs

| **Abbreviation** | **Full name** |
| --- | --- |
| CDH3 | Cadherin 3  EDA–PAA/rh Col III |
| CR1L | Complement C3b/C4b receptor 1 like |
| IGLON5 | IgLON family member 5 |
| PCDHGA11 | Protocadherin gamma subfamily A, 11 |
| FLRT2 | Fibronectin leucine rich transmembrane protein 2 |
| UNC5B | Unc-5 netrin receptor B |
| CLDN3 | Claudin 3 |
| NDNF | Neuron derived neurotrophic factor |
| TNFSF8 | TNF superfamily member 8 |
| NHSL2 | NHS like 2 |
| SGIP1 | SH3GL interacting endocytic adaptor 1 |
| SELPLG | Selectin P ligand |
| ISLR2 | Immunoglobulin superfamily containing leucine rich repeat 2 |
| BCAS1 | Brain enriched myelin associated protein 1 |
| SOX2 | SRY-box transcription factor 2 |
| FGF8 | Fibroblast growth factor 8 |
| DACH2 | Dachshund family transcription factor 2 |
| NLRP12 | NLR family pyrin domain containing 12 |
| INHBB | Inhibin subunit beta B |
| HHIP | Hedgehog interacting protein |
| TNIP3 | TNFAIP3 interacting protein 3 |
| RYR2 | Ryanodine receptor 2 |
| CTSO | Cathepsin O |
| NCF2 | Neutrophil cytosolic factor 2 |

**Table S3.** Performance comparison of mainstream clinical LAAC devices and the EDA-PAA/rhCol III coated device developed in this study

| **Device** | **Base material** | **Coating/**  **technique** | **Anti-coagulation** | **Anti-inflammation** | **Endothelialization** | **Coating Stability** |
| --- | --- | --- | --- | --- | --- | --- |
| Watchman FLX Pro | nitinol skeleton;  PET membrane | PVDF-HFP | passive effect via surface topography modulation | passive effect | no active function of pro-endothelialization | physical adhesion;  moderate stability |
| Lambre | nitinol skeleton;  PET membrane | TiN nano  -ceramic | passive effect via surface interface modulation | passive effect | no active function of pro-endothelialization | chemical bonding;  excellent stability |
| Amplatzer Amulet | nitinol skeleton;  PET membrane | no coating | no active effect | no active effect | no active function of pro-endothelialization | no coating |
| LAmax | nitinol skeleton;  PET membrane | SMART technology | active effect  via negative ion modification | passive effect | passive function of pro-endothelialization | no coating |
| This study | PLA/PDO  braided skeleton | EDA-PAA/rhCol III | active effect;  inhibits platelet  adhesion and  activation | active effect;  modulate the  anti-inflammatory phenotype | active effect;  achieving 71.68%  coverage at 40 d | covalent grafting;  good stability |
